# The Diverse Evolutionary Histories of Domesticated Metaviral Capsid Genes in Mammals

**DOI:** 10.1093/molbev/msae061

**Published:** 2024-03-20

**Authors:** William S Henriques, Janet M Young, Artem Nemudryi, Anna Nemudraia, Blake Wiedenheft, Harmit S Malik

**Affiliations:** Department of Microbiology and Cell Biology, Montana State University, Bozeman, MT 59717, USA; Basic Sciences Division, Fred Hutchinson Cancer Center, Seattle, WA 98109, USA; Department of Microbiology and Cell Biology, Montana State University, Bozeman, MT 59717, USA; Department of Microbiology and Cell Biology, Montana State University, Bozeman, MT 59717, USA; Department of Microbiology and Cell Biology, Montana State University, Bozeman, MT 59717, USA; Basic Sciences Division, Fred Hutchinson Cancer Center, Seattle, WA 98109, USA; Howard Hughes Medical Institute, Fred Hutchinson Cancer Center, Seattle, WA 98109, USA

**Keywords:** capsid, LTR retrotransposon, gene conservation, positive selection, exaptation, PNMA, SIRH, RNA-binding

## Abstract

Selfish genetic elements comprise significant fractions of mammalian genomes. In rare instances, host genomes domesticate segments of these elements for function. Using a complete human genome assembly and 25 additional vertebrate genomes, we re-analyzed the evolutionary trajectories and functional potential of capsid (CA) genes domesticated from *Metaviridae*, a lineage of retrovirus-like retrotransposons. Our study expands on previous analyses to unearth several new insights about the evolutionary histories of these ancient genes. We find that at least five independent domestication events occurred from diverse *Metaviridae*, giving rise to three universally retained single-copy genes evolving under purifying selection and two gene families unique to placental mammals, with multiple members showing evidence of rapid evolution. In the *SIRH/RTL* family, we find diverse amino-terminal domains, widespread loss of protein-coding capacity in *RTL10* despite its retention in several mammalian lineages, and differential utilization of an ancient programmed ribosomal frameshift in *RTL3* between the domesticated CA and protease domains. Our analyses also reveal that most members of the *PNMA* family in mammalian genomes encode a conserved putative amino-terminal RNA-binding domain (RBD) both adjoining and independent from domesticated CA domains. Our analyses lead to a significant correction of previous annotations of the essential *CCDC8* gene. We show that this putative RBD is also present in several extant *Metaviridae,* revealing a novel protein domain configuration in retrotransposons. Collectively, our study reveals the divergent outcomes of multiple domestication events from diverse *Metaviridae* in the common ancestor of placental mammals.

## Introduction

At least half of the human genome is derived from selfish genetic elements (Smit 1999; Li et al. 2001; Venter et al. 2001; de Koning et al. 2011). Transposons are selfish genetic elements that encode key proteins needed to replicate and spread independently of host control (Dawkins 1976; Doolittle and Sapienza 1980; Orgel and Crick 1980). In the human genome, a small number of transposons retain the capacity to “jump” to new regions of the genome, whereas the majority are found immobilized and in various stages of degradation (Venter et al. 2001; Deininger and Batzer 2002). New transposon insertions can be harmful; they can interrupt host genes, alter the expression patterns of adjacent genes, or lead to ectopic recombination (Boissinot et al. 2006). Although some new insertion events in somatic cells are implicated in disease (Burns 2017, 2020), most insertions are functionally inconsequential (Baillie et al. 2011; Fueyo et al. 2022).

Although somatic insertions in multicellular organisms are an evolutionary “dead-end”, transposon insertions in the germline are heritable. These insertions become a substrate for natural selection. Insertions that are deleterious to host fitness are rapidly eliminated from the population by purifying selection, whereas insertions that are inconsequential accumulate mutations at rates consistent with genomic mutation rates (reviewed in Mager and Stoye 2015; Wells and Feschotte 2020). However, on rare occasions, pieces of mutationally decaying transposons acquire host functions and are subsequently protected from mutational abrasion by a process known as gene domestication, exaptation, or co-option (Brandt et al. 2005b; Modzelewski et al. 2022). After acquiring host function, such transposon-derived regions are no longer capable of autonomous replication and evolve just as any other host gene subject to purifying selection.

In mammalian genomes, the most common class of selfish elements are retrotransposons, which generate new copies using a “copy-and-paste” mechanism (Burns and Boeke 2012; Finnegan 2012; Krupovic et al. 2018; Dodonova et al. 2019). Retrotransposons are classified into two broad groups: long-terminal-repeat (LTR) retrotransposons and non long-terminal-repeat (non-LTR) retrotransposons, which include both autonomous LINE-1 retrotransposons and nonautonomous Alu and SVA elements that rely on LINE-1 machinery. LTR retrotransposons include endogenous retroviruses (ERVs) (Cordaux and Batzer 2009), as well as four other families of LTR retrotransposon from the order of reverse-transcribing viruses, *Ortervirales: Metaviridae* (formerly Ty3/Gypsy)*, Caulimoviridae, Belpaoviridae,* and *Pseudoviridae* (formerly Ty1) (Krupovic et al. 2018). LTR retrotransposons encode structural group-specific antigen (*gag)* genes and enzymatic polymerase (*pol*) genes, flanked by LTRs. Their “exogenous” (transmissible) counterparts also encode an envelope protein (*env*) (Doolittle et al. 1989; Hayward 2017), which mediates membrane fusion needed for infection, and can subvert immune defenses via an immunosuppressive domain (Ashkenazi et al. 2013).

ERV genes have been repeatedly domesticated in mammals for diverse host functions, including reproduction and viral defense. The *syncytin* env-like genes of placental mammals present a spectacular example of domestication, mediating membrane fusion events needed to form multinucleated syncytial trophoblast cells in the placenta (Kim et al. 2004; Dupressoir et al. 2012). *Syncytin* gene domestication is a remarkable example of convergence, with at least seven independent events in different mammalian orders (Dupressoir et al. 2012; Lavialle et al. 2013), and in unusual lineages of lizard (Cornelis et al. 2017) and fish (Henzy et al. 2017). In addition to domestication for placental function, multiple retroviral envelope genes have also been domesticated for retroviral defense in mice (Ikeda et al. 1985; Taylor et al. 2001), humans (Frank et al. 2022), sheep (Varela et al. 2009), cats (Ito et al. 2013, 2016), and chickens (Robinson et al. 1981). These co-opted envelope genes illustrate two key concepts about gene domestication. First, the same viral protein can be independently repurposed for similar functions in different hosts. Second, domesticated copies of a single viral domain can serve diverse functions even within a single host.

The *gag* genes of reverse-transcribing viruses have been best studied in retroviruses such as HIV-1, and to a lesser extent in *Metaviridae* such as Ty3. Gag genes encode a polyprotein that includes capsid (CA), matrix (MA), and nucleocapsid (NC) domains that together package the viral genome during the virion assembly process (Dodonova et al. 2019; Olson and Musier-Forsyth 2019). Like *env*, *gag* has been domesticated both for retroviral restriction in mice (e.g. the *Fv1* gene (Bénit et al. 1997; Yap et al. 2014; Young et al. 2018)), as well as for other critical host functions (Brandt et al. 2005b; Campillos et al. 2006; Ono et al. 2006; Sekita et al. 2008; Kokošar and Kordiš 2013). At least four domesticated CA genes have been shown to assemble into CA-like structures (Pastuzyn et al. 2018; Abed et al. 2019; Erlendsson et al. 2020; Segel et al. 2021; Xu et al. 2024), which perform essential functions in host reproduction (Ono et al. 2006) and neuronal function (Nikolaienko et al. 2018; Pastuzyn et al. 2018). One of these *gag-*derived genes is vertebrate *Arc (A*ctivity-*R*egulated, *C*ytoskeletal-associated), which originated from an ancient retroelement from the clade *Metaviridae* (formerly known as Ty3/Gypsy). ARC protein forms CA-like structures and functions in the brain to regulate learning and memory as a signaling hub and messenger RNA shuttle in neurons (Nikolaienko et al. 2018; Pastuzyn et al. 2018). An independent domestication event from a distinct *Metaviridae* lineage led to *dArc1* in *Drosophila* species, which also forms CA-like structures and packages mRNA for intercellular neuronal signaling (Ashley et al. 2018; Erlendsson et al. 2020). Together, *Arc* and *dArc1* elegantly demonstrate the functional convergence of independently domesticated genes in animal lineages. Although other domesticated *gag* genes also encode proteins capable of assembling into CA-like structures, most remain functionally uncharacterized (Xu et al. 2024).

Both independent ancient *Metaviridae* domestication events and post-domestication duplications gave rise to over two dozen domesticated *gag*-like genes in the human genome, including *ARC* (Campillos et al. 2006; Kokošar and Kordiš 2013) and two small gene families, often referred to as the *PNMA* (Paraneoplastic Ma antigens) and *SIRH/RTL* (Sushi-Ichi-related Retrotransposon-Homolog/RetroTransposon-Like) families (Brandt et al. 2005b; Campillos et al. 2006; Kaneko-Ishino and Ishino 2015). Among the best characterized of these genes are *Peg10* (Paternally Expressed Gene 10, a *SIRH/RTL* family member) and *Rtl1* (retrotransposon-like 1) (Butler et al. 2001; Charlier et al. 2001; Ono et al. 2001), both of which encode proteins that contain CA domains and are essential for successful embryonic development (Ono et al. 2006; Sekita et al. 2008; Segel et al. 2021). Like ARC, PEG10 viral-like particles (VLPs) can package nucleic acid; PEG10 VLPs have been repurposed as delivery vehicles for custom nucleic acid cargos with potential therapeutic applications (Clark et al. 2007; Abed et al. 2019; Segel et al. 2021). While roles are emerging for some *PNMA* and *SIRH/RTL* genes (Foley et al. 2008; Irie et al. 2015, 2022; Lee et al. 2016; Abed et al. 2019; Fujioka et al. 2023; Ishino et al. 2023), most remain functionally uncharacterized. Similarly, in-depth evolutionary characterization has only been performed for a few of these genes, revealing a mixture of retention, and lineage-specific gene loss in some species (Brandt et al. 2005b; Irie et al. 2015, 2022; Fujioka et al. 2023), which is surprising given the essentiality of orthologs of these genes in mice.

Given the important biological functions of domesticated CA-like human genes, and their therapeutic potential, we sought a more in-depth structural and evolutionary understanding of these genes, focusing on different fates of domesticated genes. Building on previous evolutionary surveys across the family (Campillos et al. 2006; Kokošar and Kordiš 2013), and on detailed surveys of individual genes (Brandt et al. 2005b; Irie et al. 2015, 2022). We also took advantage of state-of-the-art tools for structure prediction and homology detection—AlphaFold, Foldseek, and DALI (Holm 2020; Jumper et al. 2021; van Kempen et al. 2023)—and the availability of the first telomere-to-telomere assembly of the human genome and 25 additional vertebrate genomes, to carry out a systematic bioinformatic study of the architecture, evolution, expression, and predicted protein structure of CA-derived sequences.

We found that most of the approximately two dozen *bona fide* domesticated metaviral CA-like genes show clear signatures of purifying selection. About half of these have been strictly retained across placental mammals, suggesting functions common to all lineages. Other genes have undergone lineage-specific gene duplication, pseudogenization, or loss, implying lineage-specific retention or loss of functions. Most striking among these is the *RTL10* gene, whose protein-coding capacity has only been retained in a subset of mammalian lineages. For a small subset of domesticated genes, we find evidence of positive selection, indicating their involvement in ongoing genetic conflicts or acquisition of novel functions. While re-examining the domain architecture, we revise and clarify structural features associated with domesticated genes. We find that the *RTL3* domesticated genes encode either a CA–protease (PR) or separately retained CA and PR genes in different mammalian lineages. We find no evidence for a canonical retroviral MA-like domain neighboring the CA in either the domesticated CA genes or their active metaviral relatives. Instead, we find that amino-terminal domains (NTD) are widely divergent between domesticated CA gene families, reflecting their domestication from at least four distinct *Metaviridae* families with distinct domain architectures. Among these divergent NTDs, we identify a novel, putative RNA-binding domain (RBD) encoded by the *PNMA* family, which has undergone dramatic expansion in mammalian genomes and can be retained either with or without an associated CA domain. Our study reveals that recurrent domestication of the *gag* domain from structurally diverse *Metaviridae* gave rise to genes with distinct evolutionary trajectories and structural features in placental mammals.

## Results

### Divergent Metaviral CA-derived Genes in the Human Genome

Previous analyses identified 85 human genes with homology to retroviral- or retrotransposon-encoded *gag* genes (Campillos et al. 2006), demonstrating that *gag* genes have been recurrently repurposed for host function. We updated this analysis using the newly available and complete Telomere-to-Telomere (T2T) human genome assembly (including the Y chromosome) (Nurk et al. 2022; Rhie et al. 2023). We focused on the CA domain of the *gag* gene, which shares homology across reverse-transcribing viruses (Krupovic and Koonin 2017a). To ensure the capture of remote homologs, we built Hidden Markov Models (HMMs) guided by atomic structures and AlphaFold predictions of retroviral and retrotransposon CA domains. We generated a separate HMM for each of the three clades of ERVs (*Orthoretrovirinae)* as well as *Spumavirinae* (Gifford et al. 2018) and the three major clades of LTR retrotransposons found in vertebrates: *Metaviridae* (previously known as Ty3/gypsy), *Pseudoviridae* (previously Ty1/Copia), and *Belpaoviridae* (Krupovic and Koonin 2017a; Krupovic et al. 2018) (see Methods, supplementary fig. S1 and table S1, Supplementary Material online).

Querying a six-open reading frame (ORF) translation (between stop codons) of the T2T assembly with our custom CA-specific HMMs, we identified 3,140 discrete CA-like sequences from *Orthoretrovirinae* and *Metaviridae*, but none from *Pseudoviridae*, *Belpaoviridae,* or *Spumavirinae,* consistent with previous reports (Sperber et al. 2007; Blikstad et al. 2008). Our survey found 24 of the 85 previously identified metaviral*-*derived CA genes (Campillos et al. 2006). The major reason for this disparity is that the previous study contained SCAN domains, which have been proposed to originate from ancient retrotransposon *gag* domains (Ivanov et al. 2005; Emerson and Thomas 2011; Kaneko-Ishino and Ishino 2012; Krupovic and Koonin 2017b). However, none of our HMMs for the CA domain detected the numerous known SCAN-domain-containing proteins in the human genome. To keep our analysis focused, we did not build separate HMMs for the SCAN domain.

**Fig. 1.**
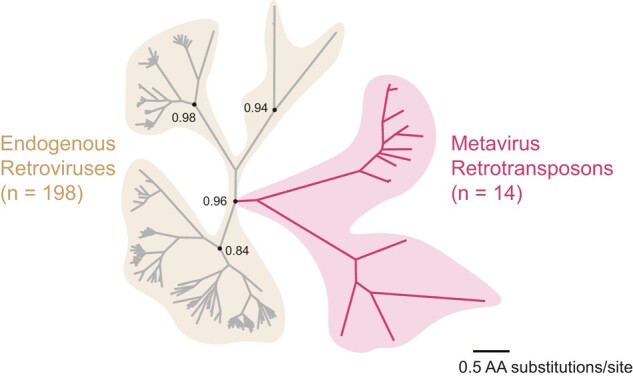
Full-length CA-like ORFs in the human genome. We generated a maximum-likelihood phylogenetic tree of 212 full-length CA-like ORFs in the human genome from an alignment of the full-length CA domain (238 positions). Hidden Markov Model profile searches identified CA sequences from ERVs (gray branches), and metavirus retrotransposons (red branches). Maximum-likelihood-based support values at selected nodes were calculated in FastTree2 (Price et al. 2010). The multiple sequence alignment and Newick files are available in the Supplementary Data, Supplementary Material online.

Of the 3,140 discrete CA-like sequences, we found only 467 CA sequences that contain both a start codon and have the potential to encode a full-length CA domain, whereas the remaining 2,673 contain premature stop codons or frameshift mutations and are unlikely to encode proteins capable of assembling into functional CAs. To understand the relationship between these 467 sequences, we aligned them to each other. This alignment allowed us to identify and remove poorly aligning sequences likely due to internal deletions, ultimately yielding 212 well-aligned open-reading frames that encode a full-length CA domain. We used this alignment to construct an unrooted maximum-likelihood phylogenetic tree (Fig. 1).

We found a notable difference in phylogenetic branching patterns between ERVs and *Metaviridae*. The majority of endogenous retroviral ORFs cluster with similar sequences related by short branches. This branching pattern is most consistent with differences that arose during recent autonomous replication (Mager and Stoye 2015). However, since it is difficult to distinguish recent insertions from evolutionarily young domesticated genes, we cannot exclude the possibility that some ERV-derived CA sequences have been recently domesticated. In contrast, the single clade representing CA-like sequences from *Metaviridae* has significantly fewer members, most of which are separated by long branches. This finding is consistent with previous analyses, which showed that *Metaviridae* ceased active transposition in an ancient ancestor of modern mammals (Brandt et al. 2005a; Blikstad et al. 2008; Mager and Stoye 2015). Thus, any remaining metaviral CA-like sequences are much more likely to be the result of host domestication (Brandt et al. 2005b; Kaneko-Ishino and Ishino 2015). Indeed, we found no adjacent long-terminal repeats flanking genomic sequences of metaviral CAs genes using LTRHarvest (Ellinghaus et al. 2008), confirming that these sequences do not represent retrotransposons capable of autonomous transposition. For this reason, we focus the remainder of our study on the 24 intact metaviral-derived CA-like sequences that remain in the human genome (we also identified two additional pseudogenes, one related to *PNMA6E/PNMA6F* and the other related to *LDOC1*). Our phylogenetic tree (Fig. 1) only contains 14 of these 24 *Metaviral* sequences because we excluded duplicates and partial CA genes. However, in subsequent analyses, we included both full-length and partial CA-like sequences because previous studies have shown that even domesticated metaviral genes encoding a truncated CA domain can still be functional (Campillos et al. 2006).

Our analyses confirm that previous catalogs of intact human-domesticated metaviral CA genes (Campillos et al. 2006; Kokošar and Kordiš 2013) were complete. These 24 human metaviral CA-like sequences correspond to almost all of the previously identified domesticated CA genes (Campillos et al. 2006; Kokošar and Kordiš 2013), including *ARC*, and members of the *PNMA* and *SIRH/RTL* gene families. The only exception is *ASPRV1* (or SASPase). A previous study identified *ASPRV1* as encoding a CA-like sequence (Campillos et al. 2006). However, a subsequent study suggested it only encodes PR but not CA domains (Kokošar and Kordiš 2013). Our analysis (both HMM analyses and AlphaFold predictions) confirms that *ASPRV1* encodes a PR domain but not a CA domain. Previous studies of the *PNMA* gene family also identified four additional members of the family that did not appear among the CA-like sequences we identified: *CCDC8, PNMA8A, PNMA8B, and PNMA8C* (Schüller et al. 2005; Pang et al. 2018). We independently identified these genes by examining sequence adjacent to CAs (see below) and included them in all subsequent analyses because of their close evolutionary ties to the CA domain in the *PNMA* family. Multiple gene nomenclatures have been adopted by different studies over the years. To avoid confusion, we list all alternative gene names to help cross-reference previous publications with our results (supplementary table S2, Supplementary Material online).

### Domesticated Metaviral CA Genes Experienced Distinct Evolutionary Retention Fates

Previous studies showed that the 24 human metaviral CA-encoding genes were domesticated in an ancient mammalian ancestor (Kaneko-Ishino and Ishino 2015) and have subsequently been conserved in at least a few mammalian species (Brandt et al. 2005b; Naruse et al. 2014; Irie et al. 2015, 2022). We investigated their evolutionary retention in a deeper sampling of representative placental mammalian genomes to identify any lineage-specific changes and to investigate their evolutionary constraints and origins. For this, we performed searches of 17 additional representative mammalian genomes (14 placental mammals, two marsupials, and one monotreme—supplementary table S3, Supplementary Material online) using sensitive HMM searches as well as iterative blast searches with both nucleotide and protein queries. We assigned the resulting sequences to orthologous groups using phylogenies and/or sequence similarity. We generated in-frame nucleotide alignments for each group (Methods, see Supplementary Materials for alignment files) and looked for inactivating frameshifts and/or premature stop codons relative to the annotated human ORF. In all cases, the full-length human annotation is well supported by conservation in distantly related placental mammal genomes (Supplementary Data sets, Supplementary Material online).

**Fig. 2.**
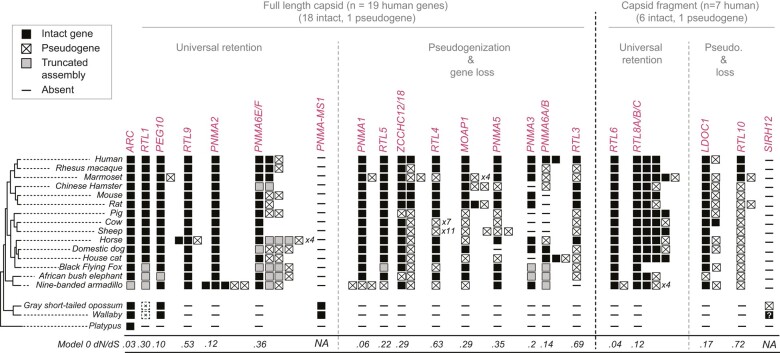
Metaviral-derived CA genes show distinct evolutionary trajectories across placental mammals. Using phylogenetic trees (supplementary figs. S2 to S7, Supplementary Material online), we assigned each metaviral-derived CA gene to one of 19 orthologous groups (labeled in red and supplementary table S2, Supplementary Material online) that are restricted to placental mammals, or to two marsupial groups (PNMA-MS1 and SIRH12). Some full-length metaviral-derived CA genes are universally retained across placental mammals as intact genes (▪), whereas others have experienced lineage-specific loss (-), pseudogenization (⊠) or duplication events (a second ▪ or ⊠ within a column). Boxes containing an “X” (i.e. ⊠) represent sequences with obvious inactivating mutations (frameshifts and/or premature stops). Gray boxes represent sequences that are truncated by gaps in the assembly. The two pseudogenes depicted for *RTL1* in opossum and wallaby (dashed squares with crosses) were previously reported as gene fragments (Edwards et al. 2008). Our analysis (performed using similar methods) did not reveal convincing *RTL1* homology in marsupial genomes. Most sequences are represented by individual boxes, but in cases where pseudogenized duplicates are numerous, the number of pseudogene duplicates is represented as *xN* (e.g. *PNMA6E/F* has four pseudogenized duplicates in horse). The status of *SIRH12* in wallaby is denoted with a “?” to indicate uncertainty. The previously reported wallaby *SIRH12* ORF (Ono et al. 2011) (with an exact match to the macEug2 version of the reference genome assembly) encodes a 107 amino acid protein, but contains a frameshifting change in a newer assembly (mMacEug1). *SIRH12*'s ortholog in opossum is a pseudogene. The previously reported *PNMA-MS2* pseudogene (Iwasaki et al. 2013) is not shown, because there are no apparently intact representatives of this sequence. The multiple sequence alignments and Newick tree files used to assign orthologous groupings are available in the Supplementary Data, Supplementary Material online.

Our phylogenomic analyses (Fig. 2, supplementary figs. S2 to S7, Supplementary Material online) confirm previous estimates of the age of these genes (Edwards et al. 2008; Iwasaki et al. 2013; Kokošar and Kordiš 2013; Kaneko-Ishino and Ishino 2015; Pastuzyn et al. 2018a). Most domesticated metaviral CA genes are at least ∼100 million years (My) old, with orthologs found in shared syntenic locations in diverse placental mammals but not in marsupials. *PEG10* is slightly older (at least ∼160 My), with additional marsupial paralogs that have been previously described (Suzuki et al. 2007; Ono et al. 2011), whereas *ARC* is much older (at least ∼350 My), with orthologs found across all tetrapods including birds, reptiles, and amphibians (Fig. 2, supplementary fig. S2, Supplementary Material online). Despite their ancient origins, the repertoire of domesticated genes has not remained static across placental mammals. We found that four orthologous groups in our trees (*PNMA6E/F, ZCCHC12/18, PNMA6A/B*, and *RTL8A/B/C*; Fig. 2, supplementary figs. S3 to S5, Supplementary Material online) contain more than one human gene (with orthologs present in other species), indicating that more recent lineage-specific duplication events continue to shape some branches of the gene family. For example, *PNMA6E* and *PNMA6F* appear to be the result of duplication in the common ancestor of primates (supplementary fig. S5, Supplementary Material online). Primates are not unusual in this regard; many other mammal lineages also appear to have undergone independent duplications in their *PNMA6E/F* family (supplementary fig. S5, Supplementary Material online). An alternative possibility is that the independent, lineage-specific *PNMA6E/F* duplications could instead be the result of recurrent, lineage-specific gene conversion events, reminiscent of the evolutionary dynamics previously described in histone variant genes (Molaro et al. 2018) and antiviral IFIT genes (Daugherty et al. 2016). After accounting for lineage-specific duplications, the 24 human-domesticated metaviral CA-like genes group into 19 clades, which likely represents the number of domesticated CA genes in the common ancestor of all placental mammals.

**Fig. 3.**
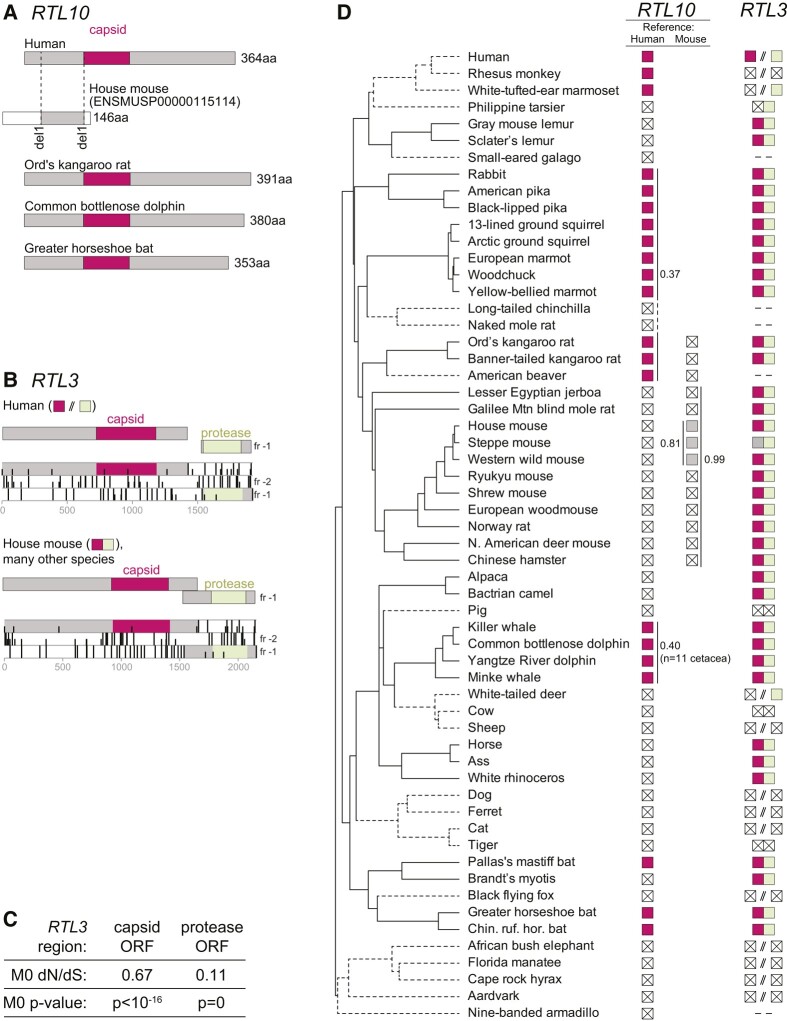
*RTL3* and *RTL10* exhibit domain-specific and lineage-specific patterns of conservation. **A**) 364aa of the CA-containing RTL10 protein are conserved in human, some rodents, cetacea, and some bats, while the annotated mouse RTL10 protein is only 146aa and the ORF has two frameshifting 1 bp deletions with respect to human *RTL10*, resulting in limited protein-coding homology and the absence of a predicted CA domain. **B)** Simplified depictions of *RTL3* showing CA homology (magenta) and PR homology in the −1 Reading frame (light green). In mouse and many other species, the two ORFs overlap and therefore likely encode a CA–PR fusion protein via a programmed ribosomal frameshift. In contrast, in human, the two ORFs do not overlap, and either encode separate proteins, or represent functional loss as seen in other simian primate genomes. Three-frame ORF analysis of *RTL3* in human and mouse showing ATG start codons (short vertical bars) and stop codons (tall vertical bars). HMM homology is shown in magenta/green, and stop-free regions containing each domain are shown in gray. **C)**  *RTL3* ORFs are subject to purifying selection for protein-coding function, with dN/dS of <1, and maximum-likelihood tests (“M0 *P*-value”) that support the dN/dS shown over the null model of neutral evolution. dN/dS values reported here differ from those reported in Figs. 2 and 4 due to the use of additional sequences. **D)** Summary of *RTL10* and *RTL3* status in an expanded set of mammalian genomes. The species tree is a trimmed version from Upham et al. (2019). Branches depicted using dashed lines show lineages where the RTL3 CA–PR fusion protein has been lost. Filled squares at the terminus of each branch represent intact ORFs and squares containing a cross represent sequences with obvious inactivating mutations (frameshifts and/or premature stops). Gray boxes represent sequences that are truncated due to genome assembly gaps, and “-”symbols indicate that we found no matching sequence. For *RTL10*, human and mouse encode different ORFs due to frameshifts: the first column (“Human”) shows that a CA-containing ORF is retained in a limited number of diverse mammalian clades, and is lost or pseudogenized in many other genomes. The second column (“Mouse”) shows that even closely related rodent genomes do not preserve the ORF found in mouse. Vertical lines with adjacent numbers show the dN/dS of these ORFs in selected clades. The multiple sequence alignments used for the PAML analyses are available in the Supplementary Data, Supplementary Material online.

Of these 19 clades, eight are universally retained as at least single intact genes across all placental mammals queried, suggesting they confer an important biological function common to all mammals (Fig. 2; *ARC*, *RTL1*, *PEG10*, *RTL9*, *PNMA2* and *PNMA6E/F*, *RTL6* and *RTL8*). Not all of these eight universally conserved genes contain full-length CA-like regions (i.e. *RTL6* and *RTL8*), confirming that a full-length CA-like domain is not necessary for the new host-related function of some of these genes (Campodonico et al. 2023). In contrast to the eight universally retained genes, we found that 11 of the domesticated full-length and fragmented CA genes that were in the common ancestor of placental mammals have since undergone gene loss or pseudogenization in one or more mammalian lineages, suggesting that their function is not universally required in mammals or is redundant with other genes (Fig. 2). For example, *RTL10* was present in the common ancestor of placental mammals but is retained as an intact ORF in only the three out of 18 mammalian genomes we surveyed. Even though *RTL10* has been repeatedly lost during mammalian evolution, deeper examination in additional species (Fig. 3A and D) reveals that *RTL10* is also retained under purifying selection as an intact gene in at least three diverse mammalian lineages: primates, basal glires, and cetaceans (see below).

Our findings are contingent on the caveats associated with reliable genome assembly. Despite significant advances since the last compendium of domesticated genes was created, most of these genome assemblies are still incomplete, leading to cases where an assembly gap results in a truncated sequence (e.g. armadillo *ARC*). This problem is compounded in the case of recent duplicates, which are likely undercounted in de novo assemblies that can collapse highly similar sequences (Fig. 2). Finally, genome assemblies contain small numbers of sequencing errors, so a minority of the apparent pseudogenes could represent intact genes with sequencing errors. Given these caveats, where possible, we used additional sequences from sister lineages to gain more confidence that inactivating mutations are not simply genome assembly or sequencing errors.

### Domesticated Metaviral CA Genes Are Retained Under Purifying and Positive Selection

**Fig. 4.**
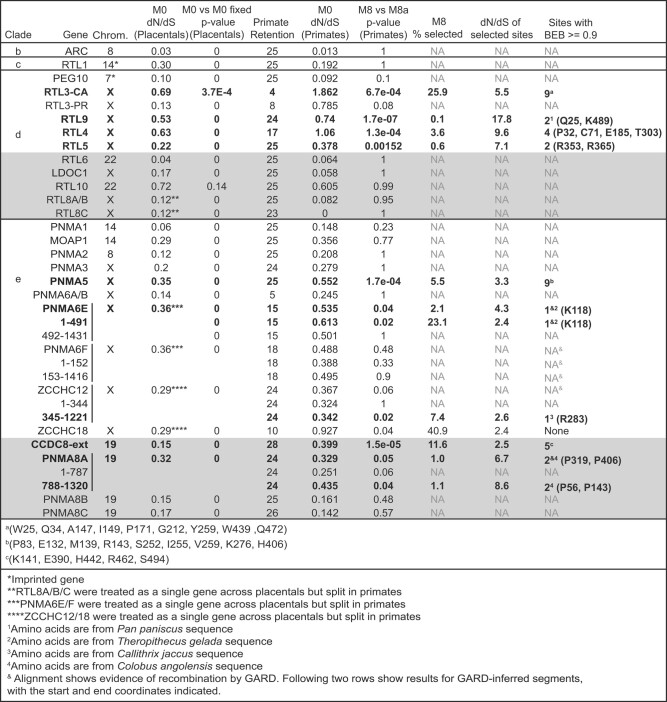
Evolutionary rates of domesticated metaviral genes across placental mammals and primates. We generated in-frame alignments for each gene across placental mammals, or across primates, and analyzed the evolutionary selective pressures on each using PAML's codeml algorithm. We used the placental mammal and primate alignments to assess “overall” selective pressure, assuming a single dN/dS ratio across sites and lineages (“M0 = model 0”). Lower dN/dS ratios indicate stronger purifying selection and dN/dS = 1 is neutral evolution. To test for positive selection, we analyzed primate alignments using PAML's codeml algorithm (codon model = 2, initial dN/dS = 0.4, cleandata = 0), but this time we compared the log likelihoods of an evolutionary model that allows for a subset of residues under positive selection (model 8) with a paired model that only allows purifying and neutral selection (models 8a). For genes where the maximum-likelihood test indicates positive selection, we report the proportion of sites estimated to be under positive selection, the dN/dS of this class of sites, and the identity of sites with a >90% posterior probability of being members of the positively selected class (codeml's “BEB” Bayes Empirical Bayes method). The multiple sequence alignments used for the PAML analysis are available in the Supplementary Data, Supplementary Material online.

The cadence and locations at which amino acid changes accumulate during evolution can also provide important clues about a protein's cellular function. We investigated the selective pressures on metaviral genes following their domestication (Fig. 2, Fig. 4), estimating the ratio of nonsynonymous (dN, amino acid altering) to synonymous (dS, amino acid preserving) nucleotide substitutions using the codeml algorithm from the PAML suite (Yang 2007). Most protein-coding genes exhibit low dN/dS (<<1), indicating purifying selection, where nonsynonymous mutations are less likely to be tolerated than synonymous mutations. dN/dS close to 1 indicates neutral evolution (no protein-coding constraint), and dN/dS > 1 indicates positive selection (adaptive evolution favors amino acid changes).

We first estimated the overall dN/dS of alignments of intact ORFs from across placental mammals (up to *n* = 18 species), using PAML's model 0 that assumes uniform selective pressures across all sites and all lineages. We compared the likelihood of this observed dN/dS to that of a model 0 with fixed dN/dS of 1, allowing us to test whether the observed dN/dS ratio was a better fit than neutral evolution. We find that most *Metaviridae*-origin CA genes evolve under overall purifying selection, with *ARC* showing the strongest purifying selection (dN/dS = 0.03). *RTL6* also shows strong purifying selection (dN/dS = 0.04), despite its involvement in immune functions (which is often associated with rapid evolution) (Irie et al. 2022).

In contrast, for *RTL10*, we were barely able to rule out the null hypothesis of neutral evolution (dN/dS = 0.72, close to the neutral ratio of 1) using our initial sampling (*n* = 3 intact primate genes). However, our deeper alignments for *RTL10* provided greater statistical power, revealing evidence of purifying selection in basal glires (rodents/lagomorphs) and in cetaceans (whales/dolphins), despite pseudogenization in other lineages (Fig. 3A and D). It remains possible that in some lineages, *RTL10* could have functions that are unrelated to its protein-coding capacity, for example as a noncoding RNA. Indeed, data from the International Mouse Phenotyping Consortium (see below) indicates that mouse knockouts of *RTL10* have both behavioral and morphological phenotypes, despite apparent frameshifting mutations in the CA-encoding region of the mouse gene (Fig. 3A). Nevertheless, *RTL10* represents an unusual case; for most domesticated genes, there is unambiguous evidence of their retention under purifying selection. Overall, our evolutionary analyses indicate that most domesticated metaviral CA genes have been retained under strong selective pressures during the last 100 million years of placental mammal evolution, suggesting that they each play important, nonredundant roles in mammalian biology.

Despite an overall signature of purifying selection, domesticated CA genes might still be subject to positive selection (dN/dS > 1) at a subset of codons as seen for host immunity-related genes such as *Fv1* (Yap et al. 2014; Young et al. 2018). To test this possibility, we collected sequences from the simian primate lineage, because this taxonomic group provides good statistical power to detect site-specific positive selection (McBee et al. 2015). We analyzed these alignments using PAML's maximum-likelihood tests that ask whether an evolutionary model that allows a subset of sites with dN/dS > 1 (NSsites model 8) is a better fit to the observed sequence data than a matched model that only allows sites under neutral and purifying selection (dN/dS of 0 to 1) (NSsites model 8a). These tests reveal that ten of these nineteen ancient metaviral genes contain a subset of sites evolving under positive selection in primates (Fig. 4), suggesting ongoing involvement in evolutionary arms races. Our findings of positive selection are intriguing given previous studies that implicate three of the positively selected genes (*CCDC8, RTL5, RTL9*) in immune-related functions (Wei et al. 2015; Irie et al. 2022; Ishino et al. 2023), suggesting that host-pathogen genetic conflict may be driving the rapid evolution of at least some of these genes.

### Domesticated CA Genes Are Structurally Diverse

**Fig. 5.**
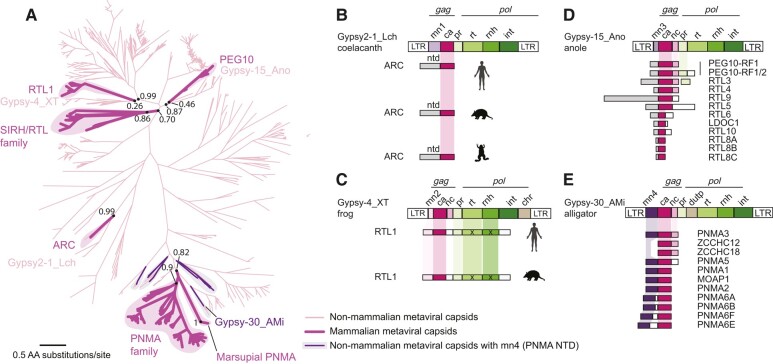
Four independent metavirus domestication events include structurally distinct N-terminal domains. **A**) A maximum-likelihood phylogenetic tree of 949 CA sequences from 24 vertebrate genomes and Repbase. 119 domesticated metaviral CA genes in mammals (dark purple, highlighted) and 830 metaviral CA-like ORFs (light pink) from selected nonmammalian vertebrate genomes: chicken (*n* = 1), alligator (*n* = 13), painted turtle (*n* = 32), anole lizard (*n* = 310), African clawed frog (*n* = 291) and coelacanth (*n* = 15) and consensus vertebrate metaviral elements from the database Repbase (*n* = 175), aligned across 172 positions in the CA domain. Maximum-likelihood-based support values calculated in FastTree2. Metaviral *gag* genes containing the PNMA *N*-terminal domain are phylogenetic neighbors to mammalian PMNA family genes (gray highlight, deep purple lines). The closest consensus Repbase sequence for each domestication is indicated (XT: *Xenopus tropicalis,* African clawed Frog; Ano: *Anolis carolensis,* anole lizard; Lch: *Latimeria chalumnae*, coelacanth; Ami: *Alligator mississippiensis*, American alligator), and **B to E)** Domain architecture (not to scale) of human *Metaviridae*-derived CA genes, organized according to major clades in the tree shown in panel A. Colored boxes indicate domains within each ORF identified by HMM profile searches, structural prediction, and structural homology searches (mn, Metaviral N-terminus, numbered 1 to 4 to indicate four unique N-terminal domains in the visualized metaviruses’ ntd, N-terminal domain; ca, capsid; nc, nucleocapsid; pr, protease; dutp, dUTPase; rt, reverse transcriptase; rnh, RNAaseH; int, integrase; chr, chromodomain; LTR, Long Terminal Repeat). The multiple sequence alignment and Newick files are available in the Supplementary Data, Supplementary Material online.

Next, we compared the domain architecture of metaviral-derived human genes to those of their metaviral progenitors, using contemporary methods for protein structure prediction and algorithms for detecting structural homology (Holm 2020; Jumper et al. 2021; van Kempen et al. 2023). To identify metaviral relatives, we queried both the Repbase database, as well as six-frame translations of nonmammalian vertebrate lineages where *Metaviridae* might be still actively retrotransposing: chicken (*Gallus gallus),* alligator (*Alligator mississippiensis),* painted turtle (*Chrysemys picta bellii)*, anole lizard (*Anolis carolensis*), African clawed frog (*Xenopus laevis*), and coelacanth (*Latimeria chalumnae*) genomes. We aligned these metaviral CA sequences with the mammalian CA-like genes and generated a maximum-likelihood phylogenetic tree of the resulting 949-sequence alignment (Fig. 5A).

Consistent with previous studies (Campillos et al. 2006; Kokošar and Kordiš 2013), our phylogeny broadly separates the domesticated metaviral genes into three groups: the *PNMA* family and the *ARC* gene (each represented by monophyletic clades), as well as a looser group containing the *SIRH/RTL* family members (including PEG10). Closer examination of the *SIRH/RTL* group subdivides that family into three monophyletic clades: *RTL1*, *PEG10*, and the remaining family members. Some previous publications have considered *RTL1* and the other *SIRH/RTL* genes as a single family (Brandt et al. 2005b; Youngson et al. 2005). However, this conclusion was based on analysis of fewer metaviral outgroup sequences. Our inclusion of a denser sampling of metaviruses reveals the independent ancestry of *RTL1, PEG10* and the remaining *SIRH/RTL* genes (Fig. 5A, supplementary fig. S7, Supplementary Material online) and is consistent with other published in-depth analyses (Campillos et al. 2006; Kokošar and Kordiš 2013). These five monophyletic clades indicate that the mammalian metaviral-derived genes derived from at least five independent domestication events. Three of these five clades are represented by single mammalian genes (*ARC, PEG10,* and *RTL1*), whereas two other clades (*PNMA* and *SIRH/RTL*) have expanded to contain many mammalian members, demonstrating that gene duplications (rather than independent retrotransposition events) probably led to the expansion of each gene family (Campillos et al. 2006; Kokošar and Kordiš 2013).

To explore the domestication events giving rise to each clade, we selected a representative consensus Repbase sequence from the nearest metaviral clades in the tree (Fig. 5A), considering these as “proxies” for the (presumed ancestral) active metaviruses that were originally domesticated. In our tree, *PEG10* and the other non*RTL1 SIRH/RTL* family members branch very closely and share the same ancestral proxy (Gypsy-15_Ano). As previously indicated, *PEG10* and the other non*RTL1 SIRH/RTL* genes likely derive from distinct domestication events since they do not form a monophyletic clade on the tree, appear to have different ages, and have a slightly different distribution, with *PEG10* also present in marsupials whereas the others are present in only placental mammals. Nevertheless, the phylogeny indicates they derived from similar, likely closely related ancestral metaviral sequences. Because of these close relationships, we treat the *PEG10* and the other non*RTL1 SIRH/RTL* genes as a single group despite their independent domestication (e.g. Fig. 5D). We emphasize that these extant metaviruses are imperfect proxies for the true ancestral viruses. For example, since PEG10 contains a classic programmed ribosomal frameshift, its metaviral ancestor almost certainly did too; however, the ancestral proxy we selected for the PEG10 clade (Gypsy-15_Ano) does not contain a frameshift.

We closely examined the domain architectures of the four “ancestral proxy” metaviral sequences and the domesticated genes that associate with each group (Fig. 5B to E). Many of the domesticated metaviral CA genes include additional domains, which sometimes include additional metaviral-derived regions, as well as protein segments with no recognizable viral homology (Campillos et al. 2006; Kokošar and Kordiš 2013). We first focused on annotating nonCA domains of “ancestral proxy” metaviruses found in our genome scan and consensus sequences from the database Repbase (Bao et al. 2015) using LTRHarvest, HMMER, PFAM (now part of Interpro), AlphaFold, Foldseek, and DALI (Ellinghaus et al. 2008; Eddy 2011; El-Gebali et al. 2019; Holm 2020; Jumper et al. 2021; van Kempen et al. 2023). We then compared the resulting patterns to those observed in the domesticated mammalian metaviral genes. For the domesticated genes, we also used these additional nonCA HMMs to search six-frame translations of 16 kb of flanking genomic sequence on each side of the CA region, to rule out the possibility of any gene mis-annotation (especially for genes that might have unannotated programmed frameshifts) or subsequent insertions having split the original metaviral domestication. Although several of our annotations agree with previously published observations (Campillos et al. 2006; Kokošar and Kordiš 2013), our analyses also reveal new insights that significantly revise the proposed domain architecture for some domesticated genes. For completeness, we both summarize previous observations as well as discuss our new findings below.

First, while many metaviral gag-derived genes encode a complete CA domain, several in the *SIRH/RTL* family encode a truncated CA domain that has only the N-terminal lobe (e.g. *LDOC1*) (Fig. 5D, supplementary fig. S6, Supplementary Material online). These truncations are seen across mammalian orthologs of each gene, indicating that they occurred soon after gene birth prior to the separation of the different mammalian orders. Notably, the AlphaFold predictions indicate that proteins encoded by these “half-CA” genes have absolutely conserved the structural fold of the N-lobe from the ancestral metavirus, whereas adjacent domains have diversified, and are typically predicted to be a mix of single alpha helices and disordered regions of varying lengths (schematized in Fig. 5, full predictions in supplementary fig. S8, Supplementary Material online). A recent report suggests the N-lobe-encoding *RTL8* regulates the function of the full-length CA-encoding *PEG10* (Campodonico et al. 2023). Other truncated CAs may perform analogous regulatory functions of full-length CA proteins.

Second, several *SIRH/RTL* family genes, as well as *RTL1*, encode full-length metaviral CA domains (and in two cases, additional domains such as NC, PR, reverse transcriptase (RT), and RNaseH) fused to long N- or C-terminal regions that have low confidence structural predictions (gray boxes, Fig. 5C and D). Our in-depth structural analysis finds no additional recognizable domains in these regions and no evidence that these regions are metaviral-derived. Although these regions are likely unstructured, they are nevertheless well conserved across placental mammalian orthologs, indicating their functional importance.

Third, *PEG10* encodes a PR domain after a −1 ribosomal frameshift that is nearly universally conserved across placental mammals; ∼30% of translating ribosomes undergo the frameshift, resulting in two different protein products: a shorter majority product containing only the *gag*-related region, and a longer minority product that also contains a PR domain (Shigemoto et al. 2001; Clark et al. 2007; Shiura et al. 2021). RTL1 proteins contain PR, and (likely catalytically inactive but structurally conserved) RT and ribonuclease H (RNase H) domains (Lynch and Tristem 2003).

Like *PEG10*, a programmed frameshift and downstream PR domain have also been suggested for the *RTL3* gene (Brandt et al. 2005a, b), although the functional relevance or evolutionarily history has not been well-defined (e.g. it is not noted in the RefSeq annotations). We used numerous additional placental mammal genomes to investigate *RTL3*'s putative programmed frameshift (Fig. 3B and D). Although *RTL3* is clearly a pseudogene in several major mammalian clades (*Carnivora*, *Bovidae*), both PR and CA-containing ORFs remain intact in many other lineages. In these instances, the PR domain is very well conserved and shows much stronger evidence of amino acid constraint (overall dN/dS = 0.11) than the CA-containing ORF (overall dN/dS = 0.67; dN/dS of CA domain alone = 0.55) (Fig. 3C). In the mouse *Rtl3* ortholog, a simple −1 frameshift would result in a fusion protein that contains both CA and PR domains. However, in human *RTL3*, a −1 frameshift would not be sufficient to produce a fusion protein, because the stop-free regions containing the two domains do not overlap and the intervening region contains stop codons in all three reading frames. Furthermore, in many other simian primate species, one or both domains of *RTL3* clearly acquired inactivating mutations in ancestral species (Fig. 3B and D). The most likely explanation is that *RTL3* encodes a CA–PR fusion protein via a programmed frameshift in mouse and many other mammalian lineages. In contrast, in human and other simian primates. *RTL3* encodes independent CA and PR proteins. Similar putative programmed frameshifts have also been reported for *PNMA3* and *PNMA5* (Wills et al. 2006). However, the sequences following the putative frameshifts are poorly conserved (unlike *PEG10* or *RTL3*) and we found no evidence for downstream functional domains; thus, their functional relevance remains unclear.

### Diverse N-terminal Structures in Domesticated Genes Derived From Metaviral Ancestors

The most surprising finding from our reannotation emerged from the analysis of the four “ancestral proxy” metaviruses, each of which has a distinct domain architecture (Fig. 5B to E). Of the four ancestral proxies, only the ARC-like metavirus lacks an NC domain immediately downstream of the CA domain. In contrast, a recognizable NC is present in the other three ancestral proxy metaviruses and has been maintained in some but not all domesticated genes in these three clades. NC domains bind viral RNAs to help package them into CA (Muriaux and Darlix 2010). Their presence in some domesticated genes suggests encoded proteins that are more likely to have RNA-binding capabilities, as has been demonstrated for PEG10 (Abed et al. 2019; Segel et al. 2021). However, ARC packages RNA even though it lacks a canonical NC domain (Pastuzyn et al. 2018). The four “ancestral proxy” metaviruses also differ in other idiosyncratic ways; a chromodomain is found only in the *RTL1* ancestor, and dUTPase only in the *PNMA* family ancestor. However, neither the chromodomain nor the dUTPase was retained in any of the domesticated genes, so we do not study these domains further.

Previous analyses have described the N-terminal region of metaviral ORFs as MA domains, by analogy to retroviral MA domains that function to facilitate membrane association and virion formation (Campillos et al. 2006; Kokošar and Kordiš 2013; Pastuzyn et al. 2018). However, this domain assignment has been based more on analogy than actual homology. Typically, retroviral MA domains fold into a globular core composed of four alpha helices with a conserved basic surface patch that facilitates membrane interaction (Murray et al. 2005; Hamard-Peron and Muriaux 2011). This helical core is also capable of binding tRNA, which regulates membrane association during virion formation (Bou-Nader et al. 2021). We explored the domain architecture of both the four “ancestral proxy” metaviruses and derived domesticated genes using a combination of BLAST, AlphaFold, DALI, and Foldseek. We were surprised to find no evidence of a canonical retroviral MA domain in any of the four metaviruses.

We found that the N-terminal domains (i.e. upstream of the CA) encoded by the *gag* gene of each of these four “ancestral proxy” metaviruses are completely dissimilar to each other and surprisingly variable in length (supplementary table S4, Supplementary Material online). According to AlphaFold, these domains are often predicted to be a single long alpha helix flanked by disordered regions (Fig. 5, supplementary table S4, Supplementary Material online, data not shown). Our analyses reveal a previously underappreciated degree of domain complexity in a poorly studied region of the *gag* gene of metaviruses and retrotransposons in general.

Given their distinct evolutionary origins and domain architecture, we examined each of the four clades of domesticated genes in more detail to infer functions. We first examined the ∼350 My-old *ARC* gene, analyzing the mammalian orthologs described above (Fig. 2, supplementary fig. S2, Supplementary Material online), as well as a more distant frog ortholog. Notably, ARC's entire gene architecture is conserved across species, not only the CA domain. However, upon the comparison of ARC with “ancestral proxy” metaviral sequences from the coelacanth, we found homology between CA domains using reciprocal HMM searches. However, N-terminal HMMs built from 11 coelacanth metaviral sequences were not homologous to N-terminal HMMs built from vertebrate ARC orthologs, which predict a ∼200 amino acids long coiled-coil domain (schematicized in Fig. 5B). Furthermore, the ARC N-terminal region HMM did not reveal matches to any metaviral retrotransposons in the Repbase database. Additional efforts using HHpred (Zimmermann et al. 2018), DALI (Holm 2020), and Foldseek (van Kempen et al. 2023) also failed to clarify the evolutionary origin of this 200 amino acid N-terminal domain of ARC. We cannot rule out the possibility that this unusual N-terminal domain derived from a metaviral lineage that does not yet have a sequenced representative, especially since the domestication event is so old. Alternatively, it remains possible that the domain was acquired from nonviral sequences during or following domestication. However, we note that no homologous matches were found in any animal genomes outside *ARC* itself. Thus, while the CA domain is unambiguously of metaviral origin, the origin of the ARC N-terminus remains mysterious and should be a focus of future analyses given its importance in ARC function (Eriksen et al. 2021).

The N-terminal regions of RTL1 and the *SIRH/RTL* family (and their closest metaviral relatives) are predicted to encode single alpha helices and disordered regions ranging from 25 residues (RTL8) to 1,169 residues (RTL9) with no clear domain architecture. The N-termini of each gene are conserved across placental orthologs but are not similar between different genes in this family, suggesting they are functionally important domains, despite our inability to predict their function through structural homology (supplementary fig. S9, Supplementary Material online). This reveals that the N-terminus of the *SIRH/RTL* family has diverged widely since domestication, whereas the CA core has retained nearly perfect structural homology to a metaviral CA domain. This contrasts starkly with the fate of the N-terminus in the *PNMA* family, which contains a highly conserved N-terminus that we discuss in more detail in the next section.

### A Novel Putative RBD in the PNMA Family

**Fig. 6.**
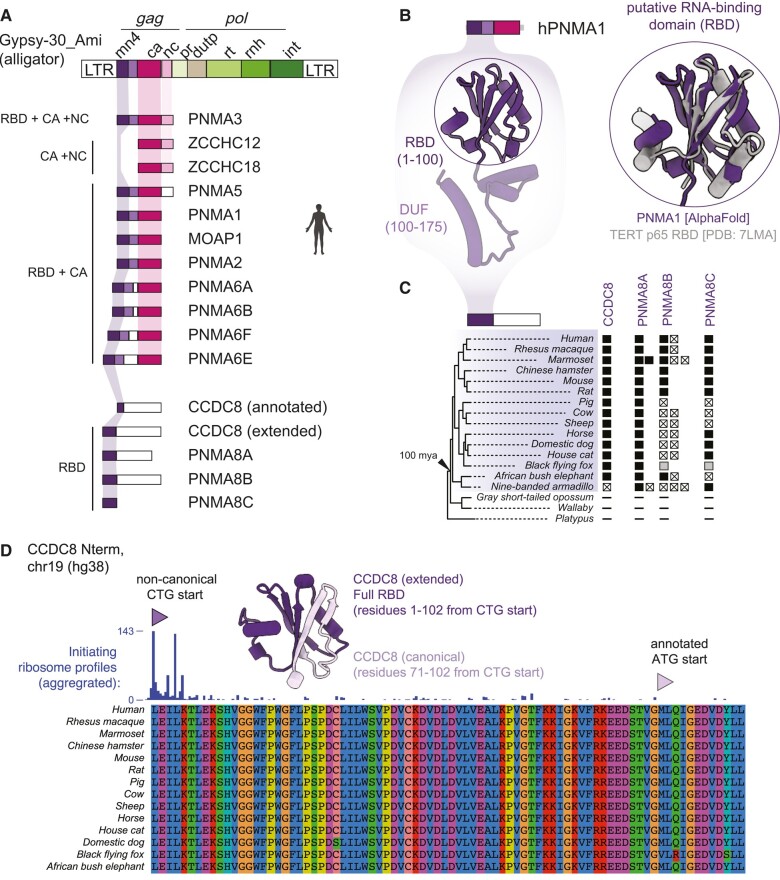
Predicted RBD in the PNMA family and related metaviruses. **A)** Illustration of a metavirus from the alligator genome colored by domain. The first ∼100 amino acids of related CAs in the human genome are predicted to form an RBD. This domain is also found independent of the CA. **B)** AlphaFold structural prediction of PNMA1 (purple), shown alone (left) as well as superimposed on an experimentally determined structure (gray, right) of the RBD (PDB: 7LMA) from telomerase p65 (RMSD between 41 pruned Cα atoms). **C)** Retention of putative RBD-only genes in placental mammals. Filled squares represent intact genes and squares containing a cross represent sequences with obvious inactivating mutations (frameshifts and/or premature stops). Gray boxes represent sequences that are truncated due to genome assembly gaps, and “-” symbols represent cases where we find no matching sequence. A known species tree is shown on the left and was obtained by pruning a whole-genome tree available via the UCSC genome browser, and **D)** CCDC8 translation initiates at an upstream noncanonical CTG start codon (dark purple). Aggregated data from many ribosomal profiling studies displayed via the GWIPS-viz genome browser (Michel et al. 2014) show an accumulation of initiating ribosomes at the noncanonical CTG start site (dark purple) rather than the canonical start site (light purple). The additional 70 N-terminal amino acids are highly conserved across mammals. The extended N-terminus is predicted (Alphafold) to encode a full-length RBD. The Alphafold predictions, multiple sequence alignments, and Newick files used to assign orthologous groups are available in the Supplementary data, Supplementary Material online.

Our structural modeling and similarity searches of the ∼175 amino acid N-terminal region in the *PNMA* clade and its closest metaviral relative revealed a predicted ∼100 amino acid RBD with mixed alpha-helical and beta sheet topology that was unrecognized in any previous analyses of either metaviral sequences or the domesticated *PNMA* gene family (Fig. 5E, Fig. 6). Using AlphaFold's structural prediction for the N-terminal domain from human PNMA1 as a query in DALI and Foldseek structural homology searches, we found high-scoring matches to RBDs found in many proteins, including an RNA recognition motif of the p65 subunit of the *Tetrahymena thermophila* telomerase complex (PDB: 7LMA) (He et al. 2021) and human MARF1 (meiosis regulator and mRNA stability factor 1, PDB: 2DGX, no structure-associated publication) (Fig. 6B). Due to several missing residues in the Gypsy-30_Ami consensus sequence, we instead used a closely related genomic sequence (also from *Alligator mississippiensis*) for structural predictions (supplementary fig. S10A, Supplementary Material online). The predicted Gypsy-30 structure has clear structural homology with human PNMA1, marsupial PNMA, and many modern metaviruses (supplementary fig. S10A, Supplementary Material online), demonstrating that this domain was present in the ancestral metavirus before domestication in mammals.

To identify all instances of human proteins that contain this novel putative RBD, we generated an HMM and performed several sensitive database searches, using a gathering threshold bit-score of 25 and a length threshold of 100 amino acids. Searching a six-frame translation of the human genome, we found that almost all *PNMA* family members, except *ZCCHC12* and *ZCCHC18,* contain a match to this RBD HMM. Nine of the human RBD-containing genes also contain a CA domain, but four do not. These four nonCA genes (*CCDC8, PNMA8A, PNMA8B* and *PNMA8C*) represent the four metaviral-derived genes that we had originally missed based on our CA-focused HMM search strategy (Fig. 6C). Thus, the only domain that links these four genes to the rest of the members of the domesticated PNMA gene family is the putative RBD, highlighting its likely functional significance. Three of these nonCA genes encode proteins in which the putative RBD is fused to unstructured C-terminal regions. We also performed the same cross-species analysis as before to investigate the evolutionary retention of this domain. We found that all *PNMA* family members, including CA-encoding and CA-lacking genes, show significant conservation of the putative RBD across placental mammals (Fig. 4, Fig. 6C, supplementary fig. S3, Supplementary Material online).

Our identification of the RBD also allowed to us to correct a major mis-annotation of one of the *PNMA* gene family members. While most of the HMM matches spanned the full length of the putative RBD, our initial survey found only a partial match to the RBD HMM in the annotated *CCDC8* protein, suggesting it contained an apparently truncated form of the domain. This truncation seemed surprising, so we explored *CCDC8* genomic sequences more closely. Using both evolutionary conservation and ribosome profiling studies that identified translation start sites, we found strong evidence that the correct initiation codon for *CCDC8* translation is a noncanonical upstream CTG start codon, encoding a 608 amino acid protein containing a full-length RBD HMM match (Fig. 6D) instead of the currently annotated 538-residue protein. Thus, identifying the novel putative RBD allowed us to correct the annotation of the essential and rapidly evolving *CCDC8* gene (Koscielny et al. 2014).

The *PNMA* gene architecture demonstrates that a metavirus containing an RBD-CA-NC domain architecture was active in an ancient mammalian ancestor. To determine how prevalent this potential RBD is in modern metaviruses, we queried Repbase using our *PNMA* family N-terminal HMM. We identified 12 consensus metaviral sequences containing this RBD domain, representing reptile, fish, and amphibian elements. We also searched six-frame translations of genomes in which metaviruses are still active: alligator (*Alligator mississippiensis),* painted turtle (*Chrysemys picta bellii)*, anole lizard (*Anolis carolensis*), African clawed frog (*Xenopus laevis*), coelacanth (*Latimeria chalumnae*), zebrafish (*Danio rerio*), and fugu (*Takifugu rubripes*) (supplementary table S3, Supplementary Material online). Our search yielded 217 RBD-like ORFs, including representatives from all genomes searched, consistent with our observations from the Repbase database. These results indicate that metaviruses with an RBD-CA-NC architecture are still actively retrotransposing in reptile, amphibian, and fish lineages. By aligning these metaviral sequences with mammalian RBD sequences and generating a maximum-likelihood phylogeny, we conclude that the domestication of RBD-like *PNMA* genes most likely occurred once in each of the common ancestors of placental mammals and marsupials, and was spread within those lineages by subsequent duplication (supplementary fig. S3, Supplementary Material online, supplementary fig. S10, Supplementary Material online), in agreement with inferences based on the CA domain (Fig. 3) (Kokošar and Kordiš 2013). Strikingly, structural predictions for this domain share a high degree of structural homology across placental mammals, marsupials, and nonmammalian vertebrates (supplementary fig. S10B, Supplementary Material online) suggesting that this protein fold is still critical to modern domesticated function. The persistence of the putative RBD in active metaviruses and its widespread conservation in domesticated PNMA-family genes highlight the importance of this domain, though its function for RNA-binding or some other function remains to be demonstrated in domesticated genes or in metaviral transposition. Thus, our detailed reanalysis of domesticated genes reveals not only an unanticipated diversity of domain architectures in metaviruses, but also an idiosyncratic lineage-specific evolutionary history of mammalian genes derived from these elements.

## Discussion

Domestication of genes derived from viruses and transposons can significantly expand the coding potential of host genomes. Our analysis of *Metaviridae-*derived CA-like genes in human and placental mammal genomes reaffirms the broad conclusions of previous studies (Campillos et al. 2006; Kaneko-Ishino and Ishino 2012, 2015; Kokošar and Kordiš 2013; Pang et al. 2018) but extends these studies with several novel conclusions. Like previous analyses, we find 24 intact human CA-containing genes derived from five independent germline integrations of ancient, diverse Metaviruses. However, we show that the evolutionary fate of these domesticated genes varies widely following domestication. Three ancient germline integrations of metaviruses created universally conserved single-copy genes (*ARC, RTL1, PEG10*), whereas the others serially duplicated to become the *SIRH/RTL* and *PNMA* gene families (Campillos et al. 2006; Kaneko-Ishino and Ishino 2012, 2015; Kokošar and Kordiš 2013). Our analysis reveals that these 24 intact human CA-containing genes and their mammalian orthologs fall into 19 orthologous groupings, likely reflecting a count of 19 domesticated CA genes in the common ancestor of placental mammals. However, a deeper examination reveals that only 8 of these 19 ancestral genes are universally conserved among placental mammals, suggesting these 8 genes perform important functions that have remained largely unchanged in ∼100 My of mammalian evolution. In contrast, other domesticated genes have experienced losses or duplications, suggesting lineage-specific function. The most extreme example of this lineage-specific retention is *RTL10*, which is only retained as a protein-coding gene in primates, basal glires, and cetaceans but lost in other mammalian lineages (Fig. 3). Several of the lineage-specific domesticated genes are known to function in placenta or the brain, both organs that exhibit distinct anatomic and functional diversity among mammals (Cho et al. 2008; Takaji et al. 2009; Irie et al. 2022). Almost all metaviral genes reveal signatures of having been preserved via purifying selection in some mammalian lineages. However, we show that some of these genes evolve under positive selection, predicting their involvement in evolutionary arms races and consistent with recent reports that some of them may carry out immune-related functions (Jiang et al. 2020; Irie et al. 2022; Ishino et al. 2023).

Domesticated metaviral genes are expressed in diverse tissues. Systematic analysis of gene expression patterns across 54 adult human tissues (Genotype-Tissue Expression Consortium, GTEx), together with placenta from the Human Protein Atlas and an independent study (Lonsdale et al. 2013; Sjöstedt et al. 2020; Gong et al. 2021) enabled us to compare expression patterns for different domesticated genes (supplementary fig. S11, Supplementary Material online). Some domesticated genes are widely expressed in all or most human tissues (e.g. *RTL8C, PNMA1*), whereas others are tissue-restricted (e.g. *PNMA6F* is expressed principally in the brain, *RTL9* principally in testis, and *RTL1* principally in the placenta). We found robust evidence of expression for all but one domesticated gene in at least one human tissue. Only *RTL4* does not exhibit robust expression in any of the tissues sampled, which could be a limitation of tissues sampled by GTEx, or because bulk RNA-seq is not sufficiently sensitive.

What are the functions of these domesticated genes? Although the organismal and cellular functions of most domesticated metaviral genes remain functionally uncharacterized, important inroads into functional characterization have been made for several metaviral genes. *ARC* has been extensively studied for its role in learning and memory (Carmichael and Henley 2018; Epstein and Finkbeiner 2018; Mabb and Ehlers 2018; Newpher et al. 2018; Nikolaienko et al. 2018; Okuno et al. 2018). *ARC* knockout mice exhibit deficits in learning, memory, and sleep (Plath et al. 2006; Manago et al. 2016; Suzuki et al. 2020). However, the exact role of the CA domain remains unclear. In addition, Kaneko-Ishino, Ishino, and colleagues have studied the function of metaviral CA genes in mice by generating knockouts of *PEG10* (Ono et al. 2006)*, RTL1* (Sekita et al. 2008; Kitazawa et al. 2017)*, LDOC1* (Naruse et al. 2014), and *RTL4 (*Irie et al. 2015*)*, *RTL9* (Ishino et al. 2023), *RTL8* (Fujioka et al. 2023), and fluorescent reporter knock-in alleles of *RTL5* and *RTL6* (Irie et al. 2022). These in-depth studies reveal that many domesticated genes have profound functional consequences on organismal fitness. For instance, loss of *PEG10* leads to complete early embryonic lethality (Ono et al. 2006), *RTL1* loss leads to partial lethality at late fetal/early neonatal stages and abnormal behavioral and musculoskeletal phenotypes in surviving animals (Sekita et al. 2008; Kitazawa et al. 2017, 2020, 2021; Chou et al. 2022), whereas loss of *LDOC1* leads to abnormal placental morphology (Naruse et al. 2014). Additional studies suggest other metaviral-derived genes function in cognition or in innate immunity in the brain (Takaji et al. 2009; Irie et al. 2015, 2022; Chou et al. 2022; Fujioka et al. 2023). Complementing these in-depth studies focused on individual genes, eight of nine knockouts of domesticated genes generated and phenotyped by the International Mouse Phenotyping Consortium reveal significant behavioral and/or physiological phenotypes (supplementary table S5, Supplementary Material online) (Koscielny et al. 2014). However, so far there is little overlap between the genes knocked out by the IMPC KO and other studies—only *RTL4* and *RTL5* have been knocked out by both the IMPC and in an independent study (Irie et al. 2015, 2022), and in these cases, results between the studies did not match. For example, Irie and colleagues were able to identify differences in noradrenaline levels in *RTL4* knockout mice that they associated with increased impulsivity, reduced attention, and memory deficits (Irie et al. 2015). However, these defects were not observed in the high throughput analyses by IMPC. Likewise, Irie and colleagues identified a role for *RTL5* in innate immunity in the mouse brain (Irie et al. 2022), but this function was not tested in the IMPC's high throughput knockout analysis.

In addition to their native endogenous functions, aberrant expression of some domesticated metaviral genes can lead to autoimmune disease. For example, *PNMA1* encodes a protein associated with the autoimmune disorder paraneoplastic syndrome (PNMA1: paraneoplastic Ma antigen 1). Other *PNMA* genes may have similar phenotypes, although it is currently unclear whether their autoimmune consequences are related to their endogenous function or the result of aberrant expression (Dalmau et al. 1999; Schüller et al. 2005; Henry 2019; Xu et al. 2024). Little is known about the endogenous cellular function of the *PNMA* family genes. Previous studies have revealed that *MOAP1* (*PNMA4)* likely plays a role in regulating apoptosis, but beyond these studies in cancer cell lines, functional studies remain sparse (Tan et al. 2001, 2005; Fu et al. 2007; Foley et al. 2008; Huang et al. 2012; Law et al. 2015). In summary, mouse knockout studies indicate that domesticated metaviral genes perform important functions; their loss leads to profoundly deleterious outcomes from embryonic lethality to cognitive deficits to organ abnormalities. These studies suggest that the domestication and subsequent proliferation of metavirus-derived genes led to novel nonredundant and essential functions (Naruse et al. 2014; Irie et al. 2015, 2022; Chou et al. 2022). However, the link between their molecular function and their metaviral origins (e.g. CA-mediated transport of RNA by ARC and PEG10) remains an active area of investigation. Our findings of lineage-specific retention of some domesticated genes further suggest that some of the functions derived from these domestication events may themselves be lineage-specific.

One of the most important contributions of our study is its novel insights into the domain architecture of *Metaviridae*-derived domesticated genes. Previous sequence homology studies showed that adjacent metaviral domains (e.g. PR RT, RNase H) have been occasionally retained alongside the CA-like region, that unstructured regions with no recognizable homology have been added to metaviral-derived sequences of some genes, and that some domesticated CA-like genes retain only one of the two CA lobes (Brandt et al. 2005b; Campillos et al. 2006; Kokošar and Kordiš 2013). Using recently developed structural prediction and homology search tools, our analysis reveals that each of the five ancient domestications has a distinct N-terminal region, with no recognizable homology to what has previously been defined as a retroviral MA domain. While the CA domain has preserved structural homology across a wide-range of reverse-transcribing viruses (Krupovic and Koonin 2017a), the N-terminus of the *gag* domain of reverse-transcribing viruses displays considerably more variability (Fig. 5, supplementary table S4, Supplementary Material online). This finding suggests that the N-terminus has been an unappreciated hotspot of evolution in the structural genes of reverse-transcribing viruses. Furthermore, our analyses reveal that one of the four ancestral viruses and most of its descendant domesticated genes (the *PNMA* family) have an N-terminal region with clear structural homology to a previously unrecognized RBDs. The putative RBD is structurally district from the MA domain typically associated with the N-terminus of the retroviral CAs, but these domains may perform a functionally analogous role, where interactions with RNA promote CA assembly and membrane association. While most *PNMA* family members retain the CA- and RBD-like domains, several have lost the CA but still contain the putative RBD. In these instances, the RBD is often fused to predicted disordered regions. Although the previous focus has been on *Metaviridae*-derived CA domains, our findings highlight a putative RBD of unknown function within the *Metaviridae gag* domain that has been captured in essential protein-coding genes in placental mammals and even amplified via gene duplication.

Our discovery of a novel RBD is especially relevant to significantly amend the annotation of *CCDC8* by identifying a noncanonical start codon that extends the N-terminal protein-coding region by 70 amino acids to now comprise an intact RBD. *CCDC8* is completely conserved in mammals, including the 70 N-terminal residues. Moreover, a knockout mouse strain from IMPC reveals an essential role for *CCDC8* at least in mice (Koscielny et al. 2014). This is important because previous experiments on *CCDC8* inadvertently relied on overexpression of a truncated *CCDC8*, which we predict would not be sufficient to recapitulate its essential function. This highlights the utility of our evolutionary and structural insights for future studies to reveal the function of one of the few essential domesticated CA genes in mammals. RBD retention in domesticated *PNMA* family genes like *CCDC8* further suggests that RNA interactions may be an important function, independent of whether they contain an adjacent CA-like region.

The *PNMA*-encoded putative RBD is present in some modern *Metaviridae* that continue to actively circulate in amphibians, reptiles, and fish. Thus, our analysis of domesticated genes not only reveals an unprecedented diversity of domain architecture in *Metaviridae*, but also identifies a previously undescribed putative RBD in vertebrate metaviral retrotransposons that has an unknown role in the metaviral lifecycle. Viral CA domains are already known to function to package viral RNA genomes, and the C-terminal NC is already predicted to bind RNAs. The prevalence of a tripartite RBD-CA-NC architecture in ancient and active metaviruses indicates that each of these domains encodes a nonredundant function, which may further facilitate metaviral CA-RNA interactions or encode other functions to potentially defend metaviral RNAs from host defenses. Thus, by capturing snapshots in time based on when they were domesticated, metaviral-derived host genes provide important archeological insights into metaviral protein domains and retrotransposition strategies. The mysterious N-terminal nonCA domain of the ARC protein may represent just such an archeological clue about an ancient domain with no extant homologs that was acquired either from *Metaviridae* or host genomes ∼350 million years ago.

## Methods

### Building CA HMMs

We queried a library of diverse CA-like sequences from *Retroviridae, Metaviridae, Pseudoviridae,* and *Belpaoviridae* (Vargiu et al. 2016; Gifford et al. 2018; Krupovic et al. 2018) using five previously generated PFAM HMMs (5 HMMs from Viral_Gag CL0148 and 10 from Gag-polyprotein CL0523 (El-Gebali et al. 2019)). However, these PFAM clans do not precisely match the CA domain. Therefore, we built new HMMs that precisely identified the CA domain in each major clade of retroviruses (*n* = 4 clades) and LTR retrotransposons (*n* = 4 clades). To build custom CA HMMs for each group of reverse-transcribing viruses, we queried NCBI's nonredundant protein database (Pruitt et al. 2007) with PFAM seed alignments (in cases where the HMM covered the full-length CA domain) or with single sequences using a single iteration of PSI-BLAST (Altschul et al. 1997). Sequences identified by PSI-BLAST were aligned in MAFFT (Rozewicki et al. 2019) and the alignment was submitted to HHpred to identify the CA domain using structural homology prediction. Alignments were trimmed to the full-length CA domain, and pressed into profile HMMs using hmmbuild from the HMMER3 package (Eddy 2011) to generate eight retrovirus or retrotransposon-specific HMMs (supplementary fig. S1A, Supplementary Material online, supplementary table S1, Supplementary Material online). Each clade of LTR retroelement is represented by a single CA HMM, except for *Metaviridae*. When our initial analysis did not identify a strong hit for ARC, we built a second metaviral HMM using previously generated ARC structures (Hallin et al. 2018).

To verify the specificity of CA HMMs, a single sequence from each class of LTR retroelement was queried with each of the eight HMMs constructed. Each HMM identified a single domain from only one of these control sequences with a high bit score and significant E-value, while returning low-scoring hits or no hit at all for CA sequences from other LTR retroelement clades (e.g. the ERV2 HMM identified the MLV CA with a bit-score of 16.1 and an e-value of 3.1e−06, while the ARC HMM identified the metaviral control CA with a bit score of 42.3 and an e-value of 3.7e−14). In sequences associated with previously determined structures (Ball et al. 2016; Qu et al. 2018; Acton et al. 2019; Nielsen et al. 2019), HMM matches overlap exactly with the bi-lobed alpha-helical CA domains. In sequences with no known molecular structure, AlphaFold (Jumper et al. 2021) was used to predict tertiary structure. In these unstudied sequences, HMMs identified domains for which AlphaFold predicted bi-lobed alpha-helical architecture (supplementary fig. S1C and D, Supplementary Material online).

### Identifying CA Genes in Vertebrate Genomes

To generate a comprehensive catalog of CA-like sequences in the human genome, we generated a six-ORF translation of the recently complete T2T genome (GCF_009914755) (Nurk et al. 2022; Rhie et al. 2023) using the EMBOSS (Rice et al. 2000) tool getorf with the following parameters: -minsize 300 (minimum ORF size 100 codons); -find 1 (return regions bounded by a start and stop codon); -table 1 (use the standard genetic code, but allowing noncanonical start codons, due to our finding that CCDC8 uses a previously unannotated CTG start codon, Fig. 6D). The resulting dataset contains 2,094,180 peptide sequences that we queried for CA homology using the previously described HMMs, using hmmscan with default parameters and with a post-scan gathering threshold bit-score of 25 and a length threshold of 125 amino acids. We repeated our search on 25 additional vertebrate genomes downloaded from the UCSC genome browser site or NCBI to identify as many metaviral CA domains as possible for our subsequent phylogeny of metaviral CA domains. We filtered matching sequences to ensure that if we had required a canonical start codon (ATG), the ORF would still be ≥300 bp in size. This approach could be less sensitive to CA domains interrupted by large insertions or introns; however, a similar search of predicted spliced transcripts found in RefSeq yielded no additional hits. Furthermore, previously annotated CA-containing domesticated genes have no introns within the CA region (Campillos et al. 2006; Kokošar and Kordiš 2013). Additionally, we downloaded Repbase 25.03 (Bao et al. 2015) and generated a six-frame translation of all sequences to identify the CA domains in previously characterized consensus sequences of vertebrate LTR retroelements.

### Matching Human CA-like ORF Sequences to Previously Annotated Genes

To determine if CA genes identified using the strategy above were previously annotated, or uncharacterized sequences, we used each sequence to retrieve query NCBI's RefSeq database using blastn, considering only exact matches. We obtained the full-length ORFs of the matching annotated genes for use in our subsequent analyses.

### Analysis of Domain Architecture and Genetic Context

To identify other metaviral fragments (protein domains or LTRs) in or adjacent to each human-domesticated metaviral CA gene, we extracted flanking nucleic acid sequences 16 kb upstream of the start codon and 16 kb downstream of the stop codon from the T2T genome assembly. These ∼33 kb genomic sequences were queried for long terminal repeats (LTRs) using LTRHarvest (Ellinghaus et al. 2008). We generated new six-frame translations of the ∼33 kb extracted sequences using getorf (see above parameters). We queried translated sequences with the entire PFAM 34.0 database to identify any recognizable protein domains nearby as well as our CA and RBD HMMs. These HMM search results allowed us to identify the conserved noncanonical start site of *CCDC8*. This noncanonical start-site was verified by visualizing Ribo-seq data for initiating ribosomes using GWIPS-vis (Michel et al. 2014).

### Phylogenetic Analysis of CA-like Sequences

To understand the evolutionary relationships between CA-like sequences in the human and 26 other vertebrate genomes, we built two initial maximum-likelihood phylogenetic trees (Fig. 1, Fig. 5A, supplementary table S3, Supplementary Material online). Full-length CA sequences were defined as ORFs longer than 150 amino acids, containing HMM alignments longer than 125 amino acids (supplementary fig. S1, Supplementary Material online). Duplicate sequences were removed using CD-Hit (sequence identity threshold flag, -c 1) (Li and Godzik 2006). Unique sequences were aligned using MAFFT (Rozewicki et al. 2019) (using the more accurate L-ins-I method). Alignments were trimmed and filtered using trimAl (gt = 0.5) (Capella-Gutiérrez et al. 2009) and visually inspected, removing any sequences containing large internal insertions and deletions. A phylogenetic tree was then constructed in Fasttree2 (Price et al. 2010) using the JTT evolutionary model with gamma-shape rate variation based on ProtTest model selection (Abascal et al. 2005). Phylogenetic trees were visualized with the ggtree package in R (Yu et al. 2017). Tree topologies were verified by building phylogenetic trees in IQTREE (Minh et al. 2020) using both UFBOOT and traditional bootstrap parameters. We found that metaviral sequences consistently separated from endogenous retroviral sequences in the human phylogeny built from 238 aligned positions (Fig. 1). Mammalian metaviral orthologs consistently separated into 4 to 5 clades with distinct groups of nonmammalian metaviral sequences as the closest-branching outgroups in a phylogenetic tree built from 172 aligned positions (Fig. 5A).

### Identification of Mammalian Orthologs

To identify potential orthologs of the human-domesticated metaviral-derived CA-like genes, we performed thorough searches of 17 additional representative mammalian genomes (14 placental mammals, two marsupials, and one monotreme—supplementary table S3, Supplementary Material online) using several complementary methods. Our process was iterative, combining multiple rounds of database searches followed by sequence analysis (alignment building and orthology checking via phylogenies).

We initiated the first search using the full-length human ORF nucleotide sequences and their translations as initial queries for tblastn searches (protein query versus nucleotide database) of predicted transcripts in the NCBI nonredundant nucleotide database, specifying the species of interest for each search. In subsequent iterations, we used our initial alignments and phylogenies (see below) to identify species/gene combinations where more attention was needed, either because a gene seemed to be missing, or because the gene prediction appeared to be truncated. To search for these “missing” genes, we employed additional strategies that are better suited for detection of degenerate pseudogenes. We added blastn searches (nucleotide query versus nucleotide database), and we searched genome assemblies rather than predicted gene sets. In some cases, we added queries from nonhuman species, choosing closely related species to the one with the missing gene. For some of the apparent pseudogenes, we added sequences from sister taxa (outside the original 18 mammalian species selected) to investigate whether inactivating mutations are real rather than genome assembly errors; seeing inactivating mutations shared across related species provides confidence they are real mutations. We complemented these blast search results by searching six-frame genome translations using our metaviral CA and putative RBD HMMs (as we did for human), ensuring that all matches were included in our final sequence collection.

We generated alignments and phylogenies to help determine orthologous relationships (depicted in Fig. 2) as well as to understand which sequences are intact genes versus pseudogenes. Blastn searches against a database of all human transcripts were also useful tools in this step, often immediately clarifying that a query sequence obviously grouped with a single human gene to the exclusion of all others. For example, using each human metaviral-derived sequence as a blast query against all human genes revealed the very high similarity between genes within each of the *PNMA6E/F*, *PNMA6A/B* and *RTL8A/B/C* groups, indicating that these human genes fall into only three clades, rather than one for each individual human gene. Using our initial blastn-based group assignments, we generated “master” in-frame nucleotide alignments, using the MACSE frame-aware alignment tool (Ranwez et al. 2018), alignments that we added to and refined during subsequent search/analysis iterations.

All except four of the HMM matches, we found among whole-genome six frame translations fit very clearly into a single master in-frame alignment. The four exceptions (bosTau9 chr3:77464558-77464911; pteAle1 NW_006435837.1:195339-195764; mMonDom1 NC_077233.1:146410251-146410589 and mMacEug1 CM051816.1:211971879-211972229) are RBD-only matches, have only remote HMM homology, and blastn searches of the RefSeq representative genomes shows that neither is preserved as an ORF outside of very closely related taxa. These sequences are therefore likely to be degenerated metaviral sequences; while they meet the criteria we defined for HMM matches, they do not appear to be true domesticated genes. One additional HMM match (mMonDom1 NC_077230.1:27205883-27206248) is clearly a recent retrotransposon insertion, as RepeatMasker recognizes it as an ERV9_MD element (ERV1 family) and finds LTRs in the flanking regions, and a blastn search reveals many almost-identical copies in the opossum genome assembly. We did not include these five sequences in our final datasets.

Analyzing our in-frame nucleotide alignments using a custom script allowed us to classify each sequence as intact (complete ORF), incomplete due to genome assembly gaps (i.e. we observed one or more N bases and/or the start/end of an assembly contig near the homologous region) or a clear pseudogene (i.e. contains frameshift and/or premature stop codons with respect to the full-length annotated human ORF, or is significantly truncated without evidence of assembly gaps). Our script makes an exception for the *PEG10* gene because it has been shown to contain a programmed ribosomal frameshift; thus, we permitted frameshifts observed within 10 codons of the expected location.

During our analyses, we noticed that NCBI's predicted transcripts are sometimes annotated to indicate that potential frameshifts and/or premature stop codons that were observed in genome assemblies have been “corrected”, with the idea they most likely represent genome sequencing errors. While this approach makes sense for genes that are rarely pseudogenized, we found that for some of these domesticated CA genes, it likely underestimates the number of true pseudogenes. For this reason, we ensure that we always used corresponding sequences extracted directly from genome assemblies, rather than the predicted transcripts in the NR database. For most apparent pseudogenes, we were able to gain additional confidence that inactivating mutations are real by adding sequences from closely related taxa, where we observed that identical inactivating mutations are often shared between taxa.

In order to more formally confirm orthology relationships, we selected full-length intact genes from all species, generated amino acid translations, and realigned them using MAFFT (Rozewicki et al. 2019). Because sequence divergence is very high between the major groups shown in Fig. 5A, we aligned sequences only within each group. We manually trimmed each alignment to contain only well-aligning regions, used ProtTest to select the best-fitting evolutionary model, and used PHYML (Guindon et al. 2010) to generate phylogenies, with 100 bootstrap replicates (JTT or LG substitution model, –pinv e –alpha e -f e). Visual inspection of the resulting trees (supplementary figs. S2 to S7, Supplementary Material online) allowed confident assignment of orthology relationships. To confirm orthology assignments for pseudogenes and recent duplicates, we also generated nucleotide-based phylogenies of alignments for individual groups, again using PHYML (parameters: GTR substitution model, flags –pinv e –alpha e -f e). We also checked that species relationships roughly followed the expected species tree (Fig. 2). Finally, we obtained gene age estimates in My using the TimeTree database (Kumar et al. 2022).

### Analysis of Evolutionary Selective Constraints

We performed two complementary analyses of the evolutionary selective pressures acting on these genes. First, we used sequences from diverse placental mammals to estimate the “overall” selective pressure on each gene. From our master in-frame nucleotide alignments for each gene, we selected only the intact genes, from only the 18 placental mammal species shown in Fig. 2. We removed any in-frame gaps from the resulting alignments, and manually trimmed each to contain only well-aligning regions. We generated a phylogeny from each trimmed alignment (using PHYML with the GTR substitution model, estimating the proportion of invariable sites, shape of the gamma distribution and nucleotide frequencies). We then used the alignment and tree as inputs to the codeml algorithm from the PAML package (Yang 2007) to estimate overall dN/dS, using “model 0”, which makes the simplifying assumption that all lineages and all amino acid sites evolve under uniform selective pressure (additional parameters: codon model = 2, initial dN/dS = 0.4, cleandata = 0). We also ran codeml under model 0 but fixing dN/dS = 1 to examine the null hypothesis that sequences evolve neutrally. We determined the level of statistical support for rejecting the null (neutral) hypothesis in favor of the alternative model where dN/dS has the value estimated by model 0 by comparing twice the difference in log-likelihoods of these two model 0 runs to a chi-squared distribution with 1 degree of freedom (Fig. 4, “M0 (Placentals)” columns).

Second, while the overall dN/dS estimates indicate that purifying selection is the prevailing evolutionary pressure operating on these genes, we wanted to explore the hypothesis that positive selection might be acting at a subset of amino acid positions in some of these genes. For this position-specific analysis, we used primate genomes, which provide a powerful clade in which to look for selection (the placental mammal alignments contain much greater diversity, such that synonymous substitutions begin to show saturation). We therefore used blastn searches of NCBI's nonredundant database to collect primate orthologs for each gene, again using reciprocal blastn searches against a local database of all human genes to ensure one-to-one orthology and selecting only intact genes for our analysis. We generated in-frame alignments using MACSE, and adjusted and trimmed alignments manually. We ran the GARD algorithm (parameters: general discrete model of site-to-site rate variation, three rate classes) on each alignment (Kosakovsky Pond et al. 2006) to check alignments for recombination, which can give rise false-positive findings of positive selection (Anisimova et al. 2003). Four alignments showed evidence of recombination (*PNMA6E*, *PNMA6F*, *PNMA8A,* and *ZCCHC12*), each with a single recombination breakpoint; in these cases we split each alignment into segments at the breakpoints indicated by GARD, and ran the following analysis on each segment separately. Again, we estimated phylogenies using PHYML (parameters as for model 0 analysis) and used PAML's codeml algorithm (codon model = 2, initial dN/dS = 0.4, cleandata = 0), but this time we compared the log likelihoods of an evolutionary model that allows for a subset of residues under positive selection (model 8) with a paired model that only allows purifying and neutral selection (models 8a). For genes where the maximum-likelihood test indicates positive selection, we report in Fig. 4 the proportion of sites estimated to be under positive selection, the dN/dS of this class of sites, and the identity of sites with a >90% posterior probability of being members of the positively selected class (codeml's “BEB” Bayes Empirical Bayes method). For these genes, we also checked the robustness of evidence favoring positive selection by re-running analyses with alternative codeml parameters (codon model = 3, starting dN/dS = 3); all positive selection findings remained consistent under alternative parameter combinations.

### Structure Predictions and Homology Modelling

To gain structural insight into the regions of human metaviral sequences not annotated by either our CA HMMs or Pfam, we downloaded all human metaviral sequences from AlphaFold's Protein Structure Database (Varadi et al. 2022) and submitted unannotated portions of each protein to DALI and Foldseek to identify structural homologs (Holm 2020; van Kempen et al. 2023). This strategy consistently identified an RBD within the N-terminus of PNMA-family proteins with hundreds of hits in DALI. For example, the N-terminus of PNMA1 identified 939 significant hits (Z-score > 2) in the PDB, the majority of which were RBDs. The top 10 hits all contained Z-scores above 6.6 for RNA-binding proteins (Fig. 6, data not shown). AlphaFold was also used to predict the structures of the closest consensus metaviral sequences from Repbase, and structural homology between metaviral sequences was determined using structural superposition (mmaker command in the ChimeraX Daily build) (Pettersen et al. 2021). This strategy confirmed the near perfect retention of CA domains in RTL, ARC, and in the PNMA family, while in the *SIRH/RTL* family only the N-terminal lobe of the CA domain is retained. This approach also revealed that most metaviral-derived genes have N-terminal regions with predicted disordered regions and no clear globular domains (Fig. 5B to E, supplementary fig. S9, Supplementary Material online).

### Phylogenetic Analysis of Putative RBD Sequences

To understand the evolutionary history of the putative RBD of the PNMA family, we built two maximum-likelihood phylogenetic trees (supplementary fig. S3 and fig. S10, Supplementary Material online). The first was built using only a subset of putative metaviral RBD sequences and all mammalian sequences to confirm orthology relationships of the mammalian PNMA genes (supplementary fig. S3, Supplementary Material online –see details in Methods section, “Identification of mammalian orthologs”). To understand the evolutionary history of the PNMA putative RBD in the context of modern metaviruses, we queried 22 vertebrate genomes (supplementary table S3, Supplementary Material online) and vertebrate metaviruses from Repbase with a single HMM built from an alignment of the first 200 amino acids of the PNMA family genes (except for ZCCHC12/18, which do not contain this N-terminus) (hmmsearch—default parameters used). This search yielded 408 full-length putative RBDs, defined as ORFs longer than 100 amino acids with bit-score > 25. Duplicate sequences were removed using cd-hit (sequence identity threshold, -c 1) (Li and Godzik 2006). Unique sequences were aligned using MAFFT (Rozewicki et al. 2019) (using the more accurate L-ins-I method). Alignments were visually inspected and any sequences containing large insertions and deletions were removed. The remaining 250 sequences were used to construct a phylogenetic in Fasttree2 (Price et al. 2010) using the JTT evolutionary model with gamma-shape rate variation based on ProtTest model selection (Abascal et al. 2005). Phylogenetic trees were visualized with the ggtree package in R (Yu et al. 2017). Alphafold predictions were made from the sequence at the indicated position on the tree, and all Alphafold predictions were subsequently aligned using the mmaker command in ChimeraX (supplementary fig. S10, Supplementary Material online).

### Expression Analysis

Tissue-specific expression data were downloaded from the Genotype-Tissue Expression (GTEx) Project, supported by the Office of the Director of the National Institutes of Health, and by NCI, NHGRI, NHLBI, NIDA, NIMH, and NINDS (Lonsdale et al. 2013). The median gene level TPM dataset was obtained from the GTEx Portal on 2021 October 13. A publicly available placental RNA-seq dataset was downloaded from the Human Protein Atlas on 2021 October 12 (https://www.proteinatlas.org/about/download) (Uhlén et al. 2015) and from (Gong et al. 2021), and fragments per kilobase per million (FPKM) values were converted to transcript per million (TPM) values. A heatmap of expression was made in R using the pheatmap package with k-means clustering turned on for columns (tissue type), but not for rows (gene) (Kolde 2019) (with flags: color = viridis(50, option = “G”, direction = −1), cutree_cols = 6, gaps_row = c(1,2, 13, 23), cluster_rows = FALSE) (supplementary fig. S11, Supplementary Material online).

### International Mouse Phenotyping Consortium

HGNC gene names were submitted as queries to the IMPC database (Koscielny et al. 2014); results are summarized in supplementary table S5, Supplementary Material online.

## Supplementary Material

msae061_Supplementary_Data

## Data Availability

All sequences used in this study are from publicly available genomic sequences (except for Repbase, which was downloaded in 2020 through a Montana State University institutional subscription). supplementary table S3, Supplementary Material online includes a list of the primary vertebrate genomes (including version) used in this study, with their respective source (either UCSC or NCBI) indicated. Coordinates from these genomes, and additional sequences used outside of these primary genomes can be found in the metadata directory of the Supplementary Data, Supplementary Material online.
