# Supplementary material for: The Diverse Evolutionary Histories of Domesticated Metaviral Capsid Genes in Mammals
Source: Mol Biol Evol. 2024 Mar 20;41(4):msae061. doi: 10.1093/molbev/msae061 (PMC11011659; doi:10.1093/molbev/msae061)
Supplement: msae061_Supplementary_Data [file msae061_supplementary_data.zip › TableS4_NtermBlastScores.pdf]

Table S4. BLAST statistics for consensus Repbase metaviral sequences used as proxies for ancestral metaviruses. Translated N-terminal domains from the start codon to the start of the capsid domain were aligned and scored using pBLAST.

	Gypsy-4	Gypsy-15	Gypsy 2-1	
Gypsy-4<br>1-68				
Gypsy-15<br>1-28	-33<br>8%<br>21%			Pairwise BLAST score<br>Percent ID<br>Percent similarity
Gypsy 2-1<br>1-196	-123<br>8%<br>17%	-155<br>6%<br>7%		Pairwise BLAST score<br>Percent ID<br>Percent similarity
Gypsy-30<br>1-100	-47<br>15%<br>28%	-70<br>8%<br>12%	-100<br>12%<br>23%	Pairwise BLAST score<br>Percent ID<br>Percent similarity
