# Supplementary material for: The Diverse Evolutionary Histories of Domesticated Metaviral Capsid Genes in Mammals
Source: Mol Biol Evol. 2024 Mar 20;41(4):msae061. doi: 10.1093/molbev/msae061 (PMC11011659; doi:10.1093/molbev/msae061)
Supplement: msae061_Supplementary_Data [file msae061_supplementary_data.zip › TableS5_phenotypes.pdf]

Table S6. Phenotypes associated with knockouts of domesticated metaviral genes in mice. P-values are reported as significant (p<0.05), not provided by the source (NP), or not applicable (NA) if no knockout has been generated.

Fig 3 Clade		Gene	Chr	KO	Zygosity	n	Tissue	Phenotype	p-value
b		ARC	8	Plath et al. 2006	-/-	5-25	brain	Disrupted memory consolidation	Sig
		ARC	8	Manago et al. 2016	-/-	6-22	brain	Disrupted social behavior, object recognition	Sig
		ARC	8	Suzuki et al. 2020	-/-	4-14	brain	Disrupted sleep regulation	Sig
c		RTL1	14*	Sekita et al. 2008	+/- (Pat. KO)	13-35	placenta	Disruption of placental development, lethal	Sig
		RTL1	14*	Kitazawa et al. 2017	+/- (Pat. KO)	16-97	placenta	Disruption of placental development, lethal	Sig
	Full CA	PEG10	7*	Ono et al. 2006	+/- (Pat. KO)	2-53	placenta	Embryonic lethal	Sig
		RTL3	X	IMPC	-/Y	8 (M)	brain	Increased freezing behavior	Sig
							brain	Increased aggression	Sig
		RTL9	X	Ishino et al. 2023	-/-	2-8	brain	Loss of antifungal immunity	NP
		RTL4	X	IMPC	-/Y	7 (F)	NA	NA	NA
		RTL4	X	Irie et al. 2015	-/Y	7 (F)	brain	Increased impulsivity, memory deficits	Sig
d		RTL5	X	IMPC	-/Y	15 (M)	eye	abnormal eye morphology	Sig
					-/Y	15 (M)	kidney	enlarged kidney	Sig
					-/Y	15 (M)	heart	enlarged heart	Sig
					-/Y	15 (M)	heart	abnormal heart morphology	Sig
					-/-	8 (F)	blood	increased circulating total protein	Sig
					-/Y	15 (M)	eye	microphthalmia	Sig
					-/Y	15 (M)	kidney	abnormal kidney morphology	Sig
					-/Y	8(M)	brain	Increased freezing behavior	Sig
		RTL5	X	Irie et. al. 2022	-/-	2-4	brain	microglial innate immunity	NP
	Truncated CA	RTL6	22	Irie et al. 2022	--	2-4	brain	microglial innate immunity	NP
		LDOC1	X	Naruse et al. 2014	-/- // -/+	3-37	placenta	Abnormal placenta, elevated progesterone	Sig
		RTL10	22	IMPC	-/-	9 (F)	brain	hyperactivity	Sig
					-/-	13 (M/F)	brain	abnormal vocalization	Sig
					-/-	13 (M/F)	skin	abnormal coat/hair pigmentation	Sig
		RTL8A/B/C	X/X/X	Fujioka et al. 2023[1]	DKO	5-14	fat tissue	Increased body weight	Sig
						5-14	brain	Disrupted social behavior	Sig
e	RBD + CA	PNMA1	14	IMPC	NP	NP	NA	None	NA
		PNMA4	14	No	NA	NA	NA	NA	NA
		PNMA2	8	IMPC	NP	NP	NA	None	NA
		PNMA3	X	IMPC	-/-	8 (F)	blood	abnormal blood uric acid levels	Sig
		PNMA5	X	No	NA	NA	NA	NA	NA
		PNMA6A	X	No	NA	NA	NA	NA	NA
		PNMA6E/F	X	No	NA	NA	NA	NA	NA
	CA	ZCCHC12	X	IMPC	NP	NP	NA	None	NA
		ZCCHC18	X	No	NA	NA	NA	NA	NA
	RBD	CCDC8-ext	19	No	NA	NA	NA	NA	NA
		CCDC8-original	19	IMPC	-/-	NA	NA	preweaning lethality, complete penetrance	Sig
					-/+	8 (M)	fat	increased total body fat amount	Sig
		PNMA8A	19	No	NA	NA	NA	NA	NA
		PNMA8B	19	No	NA	NA	NA	NA	NA
		PNMA8C	19	No	NA	NA	NA	NA	NA

*Imprinted gene
[1]bioRxiv preprint

Abbreviations:
Sig = Significant   NA = Not available
Pat = Paternal   DKO = Double knockout
NP = Not provided   IMPC = International Mouse Phenotyping Consortium
