# Supplementary material for: The Diverse Evolutionary Histories of Domesticated Metaviral Capsid Genes in Mammals
Source: Mol Biol Evol. 2024 Mar 20;41(4):msae061. doi: 10.1093/molbev/msae061 (PMC11011659; doi:10.1093/molbev/msae061)
Supplement: msae061_Supplementary_Data [file msae061_supplementary_data.zip › AllSupplementaryFigures.pdf]

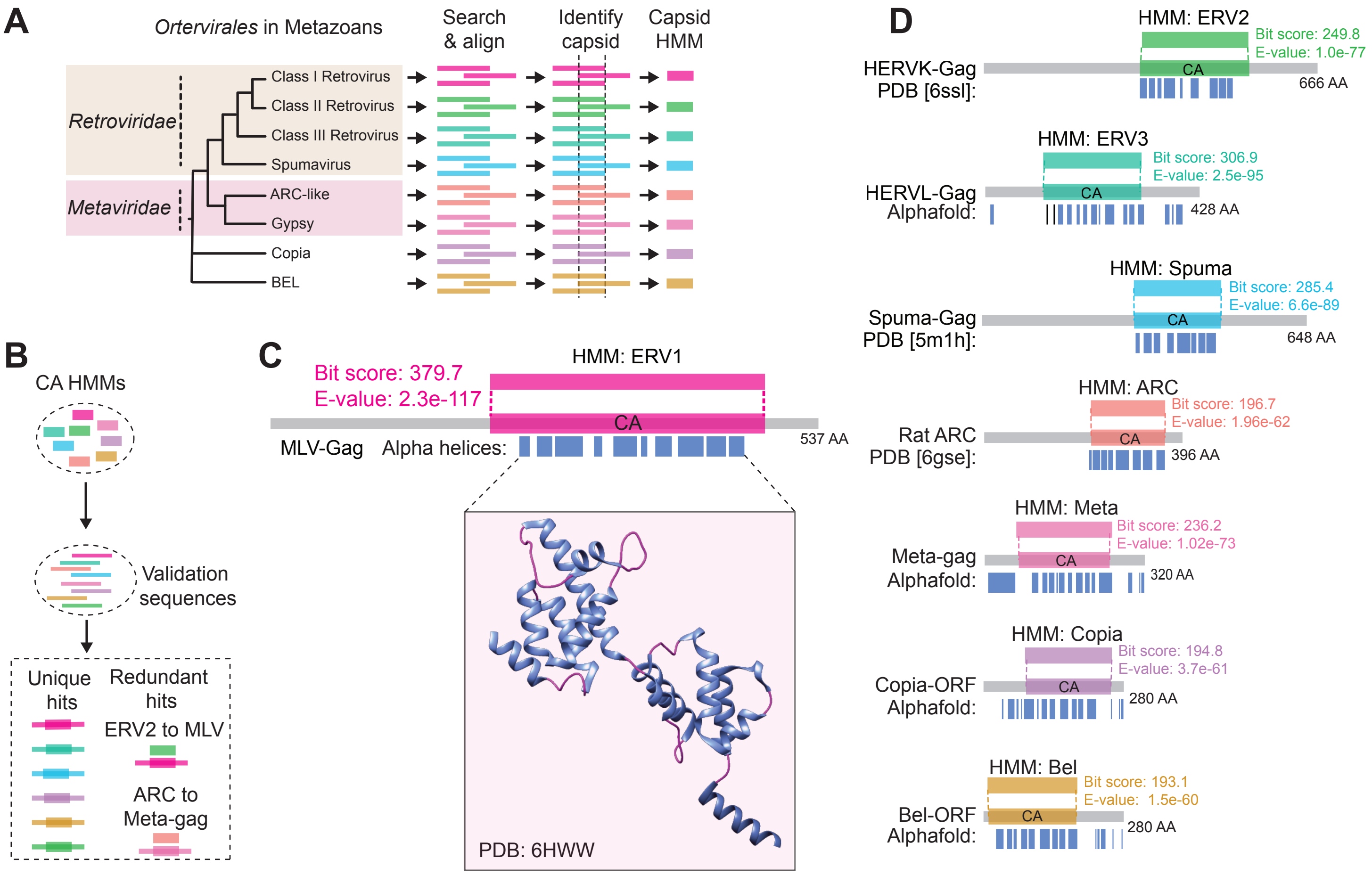

A
Ortervirales in Metazoans
Search & align
Identify capsid
Capsid HMM
Retroviridae
Metaviridae
Class I Retrovirus
Class II Retrovirus
Class III Retrovirus
Spumavirus
ARC-like
Gypsy
Copia
BEL
B
CA HMMs
Validation sequences
Unique hits
Redundant hits
ERV2 to MLV
ARC to Meta-gag
C
Bit score: 379.7
E-value: 2.3e-117
HMM: ERV1
CA
537 AA
MLV-Gag
Alpha helices:
PDB: 6HWW
D
HMM: ERV2
Bit score: 249.8
E-value: 1.0e-77
HERVK-Gag
PDB [6ssl]:
CA
666 AA
HMM: ERV3
Bit score: 306.9
E-value: 2.5e-95
HERVL-Gag
Alphafold:
428 AA
HMM: Spuma
Bit score: 285.4
E-value: 6.6e-89
Spuma-Gag
PDB [5m1h]:
648 AA
HMM: ARC
Bit score: 196.7
E-value: 1.96e-62
Rat ARC
PDB [6gse]:
396 AA
HMM: Meta
Bit score: 236.2
E-value: 1.02e-73
Meta-gag
Alphafold:
320 AA
HMM: Copia
Bit score: 194.8
E-value: 3.7e-61
Copia-ORF
Alphafold:
280 AA
HMM: Bel
Bit score: 193.1
E-value: 1.5e-60
Bel-ORF
Alphafold:
280 AA

Figure S1

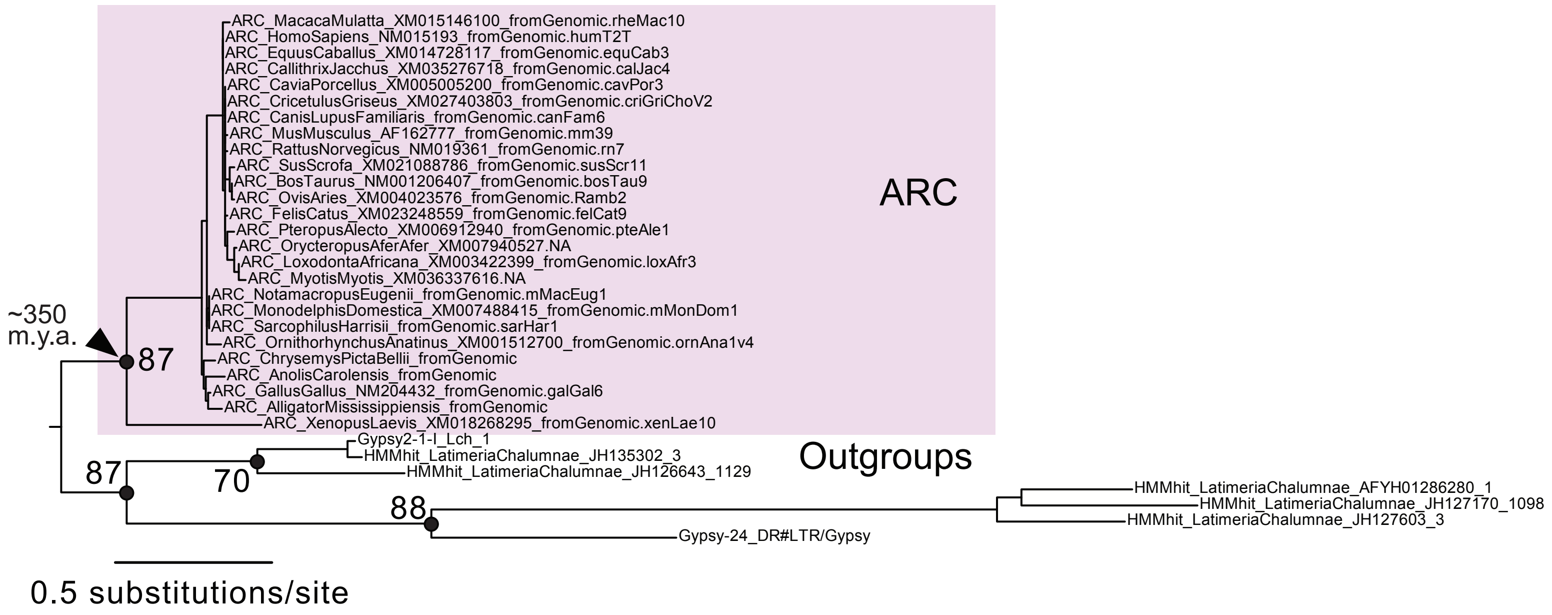
ARC_MacacaMulatta_XM015146100_fromGenomic.rheMac10
ARC_HomoSapiens_NM015193_fromGenomic.humT2T
ARC_EquusCaballus_XM014728117_fromGenomic.equCab3
ARC_CallithrixJacchus_XM035276718_fromGenomic.calJac4
ARC_CaviaPorcellus_XM005005200_fromGenomic.cavPor3
ARC_CricetulusGriseus_XM027403803_fromGenomic.criGriChoV2
ARC_CanisLupusFamiliaris_fromGenomic.canFam6
ARC_MusMusculus_AF162777_fromGenomic.mm39
ARC_RattusNorvegicus_NM019361_fromGenomic.rn7
ARC_SusScrofa_XM021088786_fromGenomic.susScr11
ARC_BosTaurus_NM001206407_fromGenomic.bosTau9
ARC_OvisAries_XM004023576_fromGenomic.Ramb2
ARC_FelisCatus_XM023248559_fromGenomic.felCat9
ARC_PteropusAlecto_XM006912940_fromGenomic.pteAle1
ARC_OrycteropusAferAfer_XM007940527.NA
ARC_LoxodontaAfricana_XM003422399_fromGenomic.loxAfr3
ARC_MyotisMyotis_XM036337616.NA
ARC_NotamacropusEugenii_fromGenomic.mMacEug1
ARC_MonodelphisDomestica_XM007488415_fromGenomic.mMonDom1
ARC_SarcophilusHarrisii_fromGenomic.sarHar1
ARC_OrnithorhynchusAnatinus_XM001512700_fromGenomic.ornAna1v4
ARC_ChrysemysPictaBellii_fromGenomic
ARC_AnolisCarolensis_fromGenomic
ARC_GallusGallus_NM204432_fromGenomic.galGal6
ARC_AlligatorMississippiensis_fromGenomic
ARC_XenopusLaevis_XM018268295_fromGenomic.xenLae10
ARC
~350
m.y.a.
87
Outgroups
Gypsy2-1-I_Lch_1
HMMhit_LatimeriaChalumnae_JH135302_3
HMMhit_LatimeriaChalumnae_JH126643_1129
87
70
88
HMMhit_LatimeriaChalumnae_AFYH01286280_1
HMMhit_LatimeriaChalumnae_JH127170_1098
HMMhit_LatimeriaChalumnae_JH127603_3
Gypsy-24_DR#LTR/Gypsy
0.5 substitutions/site

Figure S2

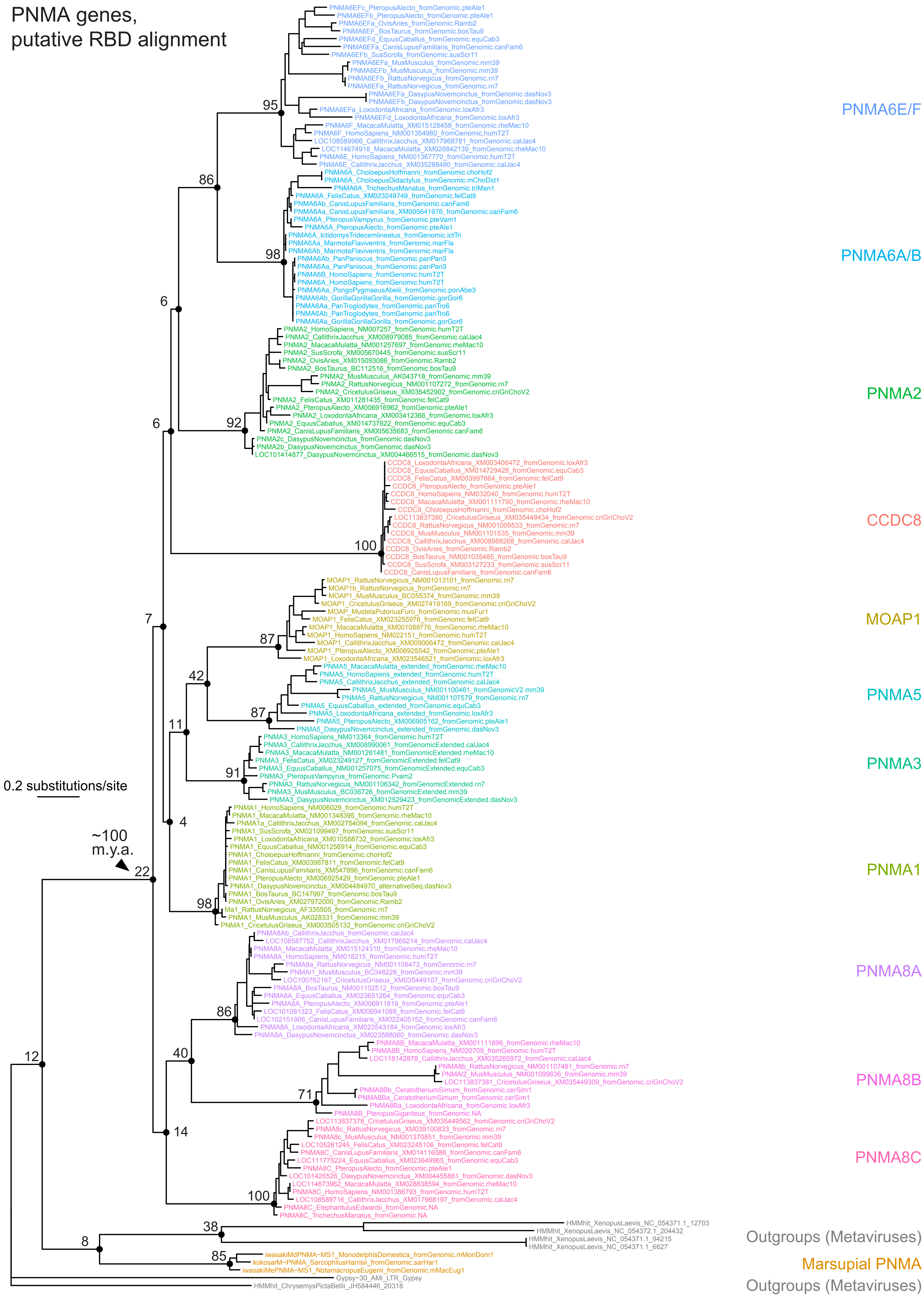
PNMA genes,
putative RBD alignment
PNMA6EFc_PteropusAlecto_fromGenomic.pteAle1
PNMA6EFb_PteropusAlecto_fromGenomic.pteAle1
PNMA6EFa_OvisAries_fromGenomic.Ramb2
PNMA6EF_BosTaurus_fromGenomic.bosTau9
PNMA6EFd_EquusCaballus_fromGenomic.equCab3
PNMA6EFa_CanisLupusFamiliaris_fromGenomic.canFam6
PNMA6EFb_SusScrofa_fromGenomic.susScr11
PNMA6EFa_MusMusculus_fromGenomic.mm39
PNMA6EFb_MusMusculus_fromGenomic.mm39
PNMA6EFb_RattusNorvegicus_fromGenomic.rn7
PNMA6EFa_RattusNorvegicus_fromGenomic.rn7
PNMA6EFa_DasypusNovemcinctus_fromGenomic.dasNov3
PNMA6EFb_DasypusNovemcinctus_fromGenomic.dasNov3
PNMA6EFa_LoxodontaAfricana_fromGenomic.loxAfr3
PNMA6EFd_LoxodontaAfricana_fromGenomic.loxAfr3
PNMA6F_MacacaMulatta_XM015128459_fromGenomic.rheMac10
PNMA6F_HomoSapiens_NM001354980_fromGenomic.humT2T
LOC108589966_CallithrixJacchus_XM017968781_fromGenomic.calJac4
LOC114674918_MacacaMulatta_XM028842139_fromGenomic.rheMac10
PNMA6E_HomoSapiens_NM001367770_fromGenomic.humT2T
PNMA6E_CallithrixJacchus_XM035288480_fromGenomic.calJac4
PNMA6E/F
95
86
PNMA6A_CholoepusHoffmanni_fromGenomic.choHof2
PNMA6A_CholoepusDidactylus_fromGenomic.mChoDid1
PNMA6A_TrichechusManatus_fromGenomic.triMan1
PNMA6A_FelisCatus_XM023249749_fromGenomic.felCat9
PNMA6Ab_CanisLupusFamiliaris_fromGenomic.canFam6
PNMA6Aa_CanisLupusFamiliaris_XM005641976_fromGenomic.canFam6
PNMA6A_PteropusVampyrus_fromGenomic.pteVam1
PNMA6A_PteropusAlecto_fromGenomic.pteAle1
PNMA6A_IctidomysTridecemlineatus_fromGenomic.ictTri
PNMA6Aa_MarmotaFlaviventris_fromGenomic.marFla
PNMA6Ab_MarmotaFlaviventris_fromGenomic.marFla
PNMA6Ab_PanPaniscus_fromGenomic.panPan3
PNMA6Aa_PanPaniscus_fromGenomic.panPan3
PNMA6B_HomoSapiens_fromGenomic.humT2T
PNMA6A_HomoSapiens_fromGenomic.humT2T
PNMA6Aa_PongoPygmaeusAbelii_fromGenomic.ponAbe3
PNMA6Ab_GorillaGorillaGorilla_fromGenomic.gorGor6
PNMA6Aa_PanTroglodytes_fromGenomic.panTro6
PNMA6Ab_PanTroglodytes_fromGenomic.panTro6
PNMA6Aa_GorillaGorillaGorilla_fromGenomic.gorGor6
PNMA6A/B
98
6
PNMA2_HomoSapiens_NM007257_fromGenomic.humT2T
PNMA2_CallithrixJacchus_XM008979085_fromGenomic.calJac4
PNMA2_MacacaMulatta_NM001257697_fromGenomic.rheMac10
PNMA2_SusScrofa_XM005670445_fromGenomic.susScr11
PNMA2_OvisAries_XM015093086_fromGenomic.Ramb2
PNMA2_BosTaurus_BC112516_fromGenomic.bosTau9
PNMA2_MusMusculus_AK043718_fromGenomic.mm39
PNMA2_RattusNorvegicus_NM001107272_fromGenomic.rn7
PNMA2_CricetulusGriseus_XM035452902_fromGenomic.criGriChoV2
PNMA2_FelisCatus_XM011281435_fromGenomic.felCat9
PNMA2_PteropusAlecto_XM006916962_fromGenomic.pteAle1
PNMA2_LoxodontaAfricana_XM003412366_fromGenomic.loxAfr3
PNMA2_EquusCaballus_XM014737822_fromGenomic.equCab3
PNMA2_CanisLupusFamiliaris_XM005635683_fromGenomic.canFam6
PNMA2c_DasypusNovemcinctus_fromGenomic.dasNov3
PNMA2b_DasypusNovemcinctus_fromGenomic.dasNov3
LOC101414877_DasypusNovemcinctus_XM004466515_fromGenomic.dasNov3
PNMA2
92
6
CCDC8_LoxodontaAfricana_XM003406472_fromGenomic.loxAfr3
CCDC8_EquusCaballus_XM014729428_fromGenomic.equCab3
CCDC8_FelisCatus_XM003997664_fromGenomic.felCat9
CCDC8_PteropusAlecto_fromGenomic.pteAle1
CCDC8_HomoSapiens_NM032040_fromGenomic.humT2T
CCDC8_MacacaMulatta_XM001111790_fromGenomic.rheMac10
CCDC8_CholoepusHoffmanni_fromGenomic.choHof2
LOC113837380_CricetulusGriseus_XM035449434_fromGenomic.criGriChoV2
CCDC8_RattusNorvegicus_NM001009533_fromGenomic.rn7
CCDC8_MusMusculus_NM001101535_fromGenomic.mm39
CCDC8_CallithrixJacchus_XM008988268_fromGenomic.calJac4
CCDC8_OvisAries_fromGenomic.Ramb2
CCDC8_BosTaurus_NM001035485_fromGenomic.bosTau9
CCDC8_SusScrofa_XM003127233_fromGenomic.susScr11
CCDC8_CanisLupusFamiliaris_fromGenomic.canFam6
CCDC8
100
7
MOAP1_RattusNorvegicus_NM001013101_fromGenomic.rn7
MOAP1b_RattusNorvegicus_fromGenomic.rn7
MOAP1_MusMusculus_BC055374_fromGenomic.mm39
MOAP1_CricetulusGriseus_XM027419169_fromGenomic.criGriChoV2
MOAP_MustelaPutoriusFuro_fromGenomic.musFur1
MOAP1_FelisCatus_XM023255976_fromGenomic.felCat9
MOAP1_MacacaMulatta_XM001089776_fromGenomic.rheMac10
MOAP1_HomoSapiens_NM022151_fromGenomic.humT2T
MOAP1_CallithrixJacchus_XM009006472_fromGenomic.calJac4
MOAP1_PteropusAlecto_XM006925542_fromGenomic.pteAle1
MOAP1_LoxodontaAfricana_XM023546521_fromGenomic.loxAfr3
MOAP1
87
42
PNMA5_MacacaMulatta_extended_fromGenomic.rheMac10
PNMA5_HomoSapiens_extended_fromGenomic.humT2T
PNMA5_CallithrixJacchus_extended_fromGenomic.calJac4
PNMA5_MusMusculus_NM001100461_fromGenomicV2.mm39
PNMA5_RattusNorvegicus_NM001107579_fromGenomic.rn7
PNMA5_EquusCaballus_extended_fromGenomic.equCab3
PNMA5_LoxodontaAfricana_extended_fromGenomic.loxAfr3
PNMA5_PteropusAlecto_XM006905162_fromGenomic.pteAle1
PNMA5_DasypusNovemcinctus_extended_fromGenomic.dasNov3
PNMA5
87
11
PNMA3_HomoSapiens_NM013364_fromGenomic.humT2T
PNMA3_CallithrixJacchus_XM008990061_fromGenomicExtended.calJac4
PNMA3_MacacaMulatta_NM001261481_fromGenomicExtended.rheMac10
PNMA3_FelisCatus_XM023249127_fromGenomicExtended.felCat9
PNMA3_EquusCaballus_NM001257075_fromGenomicExtended.equCab3
PNMA3_PteropusVampyrus_fromGenomic.Pvam2
PNMA3_RattusNorvegicus_NM001106342_fromGenomicExtended.rn7
PNMA3_MusMusculus_BC036726_fromGenomicExtended.mm39
PNMA3_DasypusNovemcinctus_XM012529423_fromGenomicExtended.dasNov3
PNMA3
91
0.2 substitutions/site
4
PNMA1_HomoSapiens_NM006029_fromGenomic.humT2T
PNMA1_MacacaMulatta_NM001348395_fromGenomic.rheMac10
PNMA1a_CallithrixJacchus_XM002754094_fromGenomic.calJac4
PNMA1_SusScrofa_XM021099497_fromGenomic.susScr11
PNMA1_LoxodontaAfricana_XM010588732_fromGenomic.loxAfr3
PNMA1_EquusCaballus_NM001256914_fromGenomic.equCab3
PNMA1_CholoepusHoffmanni_fromGenomic.choHof2
PNMA1_FelisCatus_XM003987811_fromGenomic.felCat9
PNMA1_CanisLupusFamiliaris_XM547896_fromGenomic.canFam6
PNMA1_PteropusAlecto_XM006925429_fromGenomic.pteAle1
PNMA1_DasypusNovemcinctus_XM004484970_alternativeSeq.dasNov3
PNMA1_BosTaurus_BC147997_fromGenomic.bosTau9
PNMA1_OvisAries_XM027972000_fromGenomic.Ramb2
Ma1_RattusNorvegicus_AF335505_fromGenomic.rn7
PNMA1_MusMusculus_AK028331_fromGenomic.mm39
PNMA1_CricetulusGriseus_XM003505132_fromGenomic.criGriChoV2
PNMA1
~100
m.y.a.
22
98
PNMA8Ab_CallithrixJacchus_fromGenomic.calJac4
LOC108587752_CallithrixJacchus_XM017969214_fromGenomic.calJac4
PNMA8A_MacacaMulatta_XM015124310_fromGenomic.rheMac10
PNMA8A_HomoSapiens_NM018215_fromGenomic.humT2T
PNMA8a_RattusNorvegicus_NM001108473_fromGenomic.rn7
PNMAl1_MusMusculus_BC048228_fromGenomic.mm39
LOC100762167_CricetulusGriseus_XM035449107_fromGenomic.criGriChoV2
PNMA8A_BosTaurus_NM001102512_fromGenomic.bosTau9
PNMA8A_EquusCaballus_XM023651264_fromGenomic.equCab3
PNMA8A_PteropusAlecto_XM006911819_fromGenomic.pteAle1
LOC101091323_FelisCatus_XM006941089_fromGenomic.felCat9
LOC102151906_CanisLupusFamiliaris_XM022405152_fromGenomic.canFam6
PNMA8A_LoxodontaAfricana_XM023543184_fromGenomic.loxAfr3
PNMA8A_DasypusNovemcinctus_XM023588060_fromGenomic.dasNov3
PNMA8A
86
40
PNMA8B_MacacaMulatta_XM001111896_fromGenomic.rheMac10
PNMA8B_HomoSapiens_NM020709_fromGenomic.humT2T
LOC118142878_CallithrixJacchus_XM035285972_fromGenomic.calJac4
PNMA8b_RattusNorvegicus_NM001107481_fromGenomic.rn7
PNMAl2_MusMusculus_NM001099636_fromGenomic.mm39
LOC113837381_CricetulusGriseus_XM035449105_fromGenomic.criGriChoV2
PNMA8Bb_CeratotheriumSimum_fromGenomic.cerSim1
PNMA8Ba_CeratotheriumSimum_fromGenomic.cerSim1
PNMA8Ba_LoxodontaAfricana_fromGenomic.loxAfr3
PNMA8B_PteropusGiganteus_fromGenomic.NA
PNMA8B
71
12
14
LOC113837378_CricetulusGriseus_XM035449562_fromGenomic.criGriChoV2
PNMA8c_RattusNorvegicus_XM039100833_fromGenomic.rn7
PNMA8c_MusMusculus_NM001370851_fromGenomic.mm39
LOC105261245_FelisCatus_XM023245106_fromGenomic.felCat9
PNMA8C_CanisLupusFamiliaris_XM014116586_fromGenomic.canFam6
LOC111775224_EquusCaballus_XM023649965_fromGenomic.equCab3
PNMA8C_PteropusAlecto_fromGenomic.pteAle1
LOC101426526_DasypusNovemcinctus_XM004455881_fromGenomic.dasNov3
LOC114673962_MacacaMulatta_XM028838594_fromGenomic.rheMac10
PNMA8C_HomoSapiens_NM001386793_fromGenomic.humT2T
LOC108589716_CallithrixJacchus_XM017968197_fromGenomic.calJac4
PNMA8C_ElephantulusEdwardii_fromGenomic.NA
PNMA8C_TrichechusManatus_fromGenomic.NA
PNMA8C
100
38
HMMhit_XenopusLaevis_NC_054371.1_12703
HMMhit_XenopusLaevis_NC_054372.1_204432
HMMhit_XenopusLaevis_NC_054371.1_94215
HMMhit_XenopusLaevis_NC_054371.1_6627
Outgroups (Metaviruses)
8
85
iwasakiMdPNMA−MS1_MonodelphisDomestica_fromGenomic.mMonDom1
kokosarM−PNMA_SarcophilusHarrisii_fromGenomic.sarHar1
iwasakiMePNMA−MS1_NotamacropusEugenii_fromGenomic.mMacEug1
Marsupial PNMA
Gypsy−30_AMi_LTR_Gypsy
HMMhit_ChrysemysPictaBellii_JH584446_20318
Outgroups (Metaviruses)

Figure S3

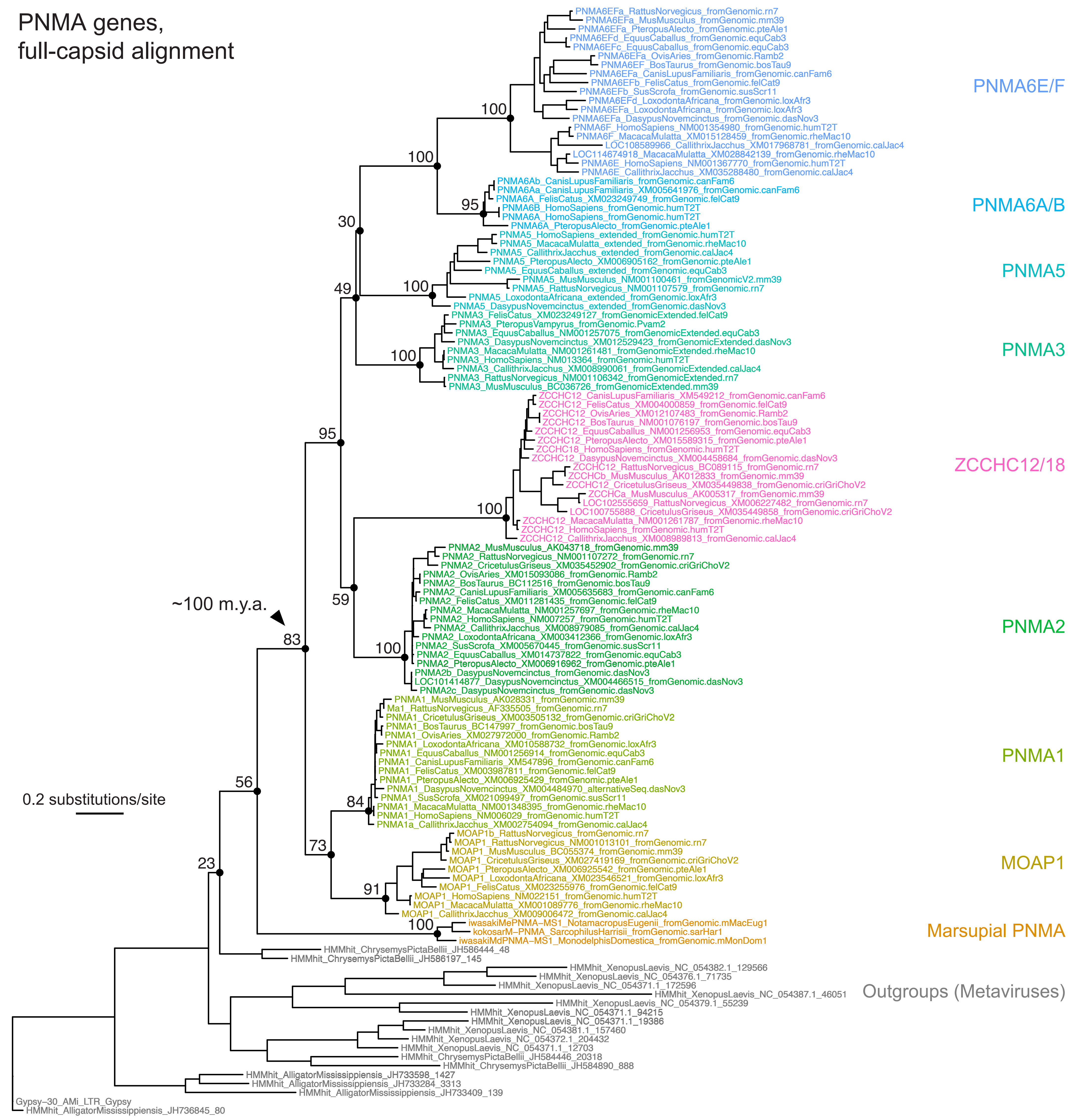

PNMA genes,
full-capsid alignment
PNMA6EFa_RattusNorvegicus_fromGenomic.rn7
PNMA6EFa_MusMusculus_fromGenomic.mm39
PNMA6EFa_PteropusAlecto_fromGenomic.pteAle1
PNMA6EFd_EquusCaballus_fromGenomic.equCab3
PNMA6EFc_EquusCaballus_fromGenomic.equCab3
PNMA6EFa_OvisAries_fromGenomic.Ramb2
PNMA6EF_BosTaurus_fromGenomic.bosTau9
PNMA6EFa_CanisLupusFamiliaris_fromGenomic.canFam6
PNMA6EFb_FelisCatus_fromGenomic.felCat9
PNMA6EFb_SusScrofa_fromGenomic.susScr11
PNMA6EFd_LoxodontaAfricana_fromGenomic.loxAfr3
PNMA6EFa_LoxodontaAfricana_fromGenomic.loxAfr3
PNMA6EFa_DasypusNovemcinctus_fromGenomic.dasNov3
PNMA6F_HomoSapiens_NM001354980_fromGenomic.humT2T
PNMA6F_MacacaMulatta_XM015128459_fromGenomic.rheMac10
LOC108589966_CallithrixJacchus_XM017968781_fromGenomic.calJac4
LOC114674918_MacacaMulatta_XM028842139_fromGenomic.rheMac10
PNMA6E_HomoSapiens_NM001367770_fromGenomic.humT2T
PNMA6E_CallithrixJacchus_XM035288480_fromGenomic.calJac4
PNMA6E/F
PNMA6Ab_CanisLupusFamiliaris_fromGenomic.canFam6
PNMA6Aa_CanisLupusFamiliaris_XM005641976_fromGenomic.canFam6
PNMA6A_FelisCatus_XM023249749_fromGenomic.felCat9
PNMA6B_HomoSapiens_fromGenomic.humT2T
PNMA6A_HomoSapiens_fromGenomic.humT2T
PNMA6A_PteropusAlecto_fromGenomic.pteAle1
PNMA6A/B
PNMA5_HomoSapiens_extended_fromGenomic.humT2T
PNMA5_MacacaMulatta_extended_fromGenomic.rheMac10
PNMA5_CallithrixJacchus_extended_fromGenomic.calJac4
PNMA5_PteropusAlecto_XM006905162_fromGenomic.pteAle1
PNMA5_EquusCaballus_extended_fromGenomic.equCab3
PNMA5_MusMusculus_NM001100461_fromGenomicV2.mm39
PNMA5_RattusNorvegicus_NM001107579_fromGenomic.rn7
PNMA5_LoxodontaAfricana_extended_fromGenomic.loxAfr3
PNMA5_DasypusNovemcinctus_extended_fromGenomic.dasNov3
PNMA5
PNMA3_FelisCatus_XM023249127_fromGenomicExtended.felCat9
PNMA3_PteropusVampyrus_fromGenomic.Pvam2
PNMA3_EquusCaballus_NM001257075_fromGenomicExtended.equCab3
PNMA3_DasypusNovemcinctus_XM012529423_fromGenomicExtended.dasNov3
PNMA3_MacacaMulatta_NM001261481_fromGenomicExtended.rheMac10
PNMA3_HomoSapiens_NM013364_fromGenomic.humT2T
PNMA3_CallithrixJacchus_XM008990061_fromGenomicExtended.calJac4
PNMA3_RattusNorvegicus_NM001106342_fromGenomicExtended.rn7
PNMA3_MusMusculus_BC036726_fromGenomicExtended.mm39
PNMA3
ZCCHC12_CanisLupusFamiliaris_XM549212_fromGenomic.canFam6
ZCCHC12_FelisCatus_XM004000859_fromGenomic.felCat9
ZCCHC12_OvisAries_XM012107483_fromGenomic.Ramb2
ZCCHC12_BosTaurus_NM001076197_fromGenomic.bosTau9
ZCCHC12_EquusCaballus_NM001256953_fromGenomic.equCab3
ZCCHC12_PteropusAlecto_XM015589315_fromGenomic.pteAle1
ZCCHC18_HomoSapiens_fromGenomic.humT2T
ZCCHC12_DasypusNovemcinctus_XM004458684_fromGenomic.dasNov3
ZCCHC12_RattusNorvegicus_BC089115_fromGenomic.rn7
ZCCHCb_MusMusculus_AK012833_fromGenomic.mm39
ZCCHC12_CricetulusGriseus_XM035449838_fromGenomic.criGriChoV2
ZCCHCa_MusMusculus_AK005317_fromGenomic.mm39
LOC102555659_RattusNorvegicus_XM006227482_fromGenomic.rn7
LOC100755888_CricetulusGriseus_XM035449858_fromGenomic.criGriChoV2
ZCCHC12_MacacaMulatta_NM001261787_fromGenomic.rheMac10
ZCCHC12_HomoSapiens_fromGenomic.humT2T
ZCCHC12_CallithrixJacchus_XM008989813_fromGenomic.calJac4
ZCCHC12/18
PNMA2_MusMusculus_AK043718_fromGenomic.mm39
PNMA2_RattusNorvegicus_NM001107272_fromGenomic.rn7
PNMA2_CricetulusGriseus_XM035452902_fromGenomic.criGriChoV2
PNMA2_OvisAries_XM015093086_fromGenomic.Ramb2
PNMA2_BosTaurus_BC112516_fromGenomic.bosTau9
PNMA2_CanisLupusFamiliaris_XM005635683_fromGenomic.canFam6
PNMA2_FelisCatus_XM011281435_fromGenomic.felCat9
PNMA2_MacacaMulatta_NM001257697_fromGenomic.rheMac10
PNMA2_HomoSapiens_NM007257_fromGenomic.humT2T
PNMA2_CallithrixJacchus_XM008979085_fromGenomic.calJac4
PNMA2_LoxodontaAfricana_XM003412366_fromGenomic.loxAfr3
PNMA2_SusScrofa_XM005670445_fromGenomic.susScr11
PNMA2_EquusCaballus_XM014737822_fromGenomic.equCab3
PNMA2_PteropusAlecto_XM006916962_fromGenomic.pteAle1
PNMA2b_DasypusNovemcinctus_fromGenomic.dasNov3
LOC101414877_DasypusNovemcinctus_XM004466515_fromGenomic.dasNov3
PNMA2c_DasypusNovemcinctus_fromGenomic.dasNov3
PNMA2
~100 m.y.a.
PNMA1_MusMusculus_AK028331_fromGenomic.mm39
Ma1_RattusNorvegicus_AF335505_fromGenomic.rn7
PNMA1_CricetulusGriseus_XM003505132_fromGenomic.criGriChoV2
PNMA1_BosTaurus_BC147997_fromGenomic.bosTau9
PNMA1_OvisAries_XM027972000_fromGenomic.Ramb2
PNMA1_LoxodontaAfricana_XM010588732_fromGenomic.loxAfr3
PNMA1_EquusCaballus_NM001256914_fromGenomic.equCab3
PNMA1_CanisLupusFamiliaris_XM547896_fromGenomic.canFam6
PNMA1_FelisCatus_XM003987811_fromGenomic.felCat9
PNMA1_PteropusAlecto_XM006925429_fromGenomic.pteAle1
PNMA1_DasypusNovemcinctus_XM004484970_alternativeSeq.dasNov3
PNMA1_SusScrofa_XM021099497_fromGenomic.susScr11
PNMA1_MacacaMulatta_NM001348395_fromGenomic.rheMac10
PNMA1_HomoSapiens_NM006029_fromGenomic.humT2T
PNMA1a_CallithrixJacchus_XM002754094_fromGenomic.calJac4
PNMA1
0.2 substitutions/site
MOAP1b_RattusNorvegicus_fromGenomic.rn7
MOAP1_RattusNorvegicus_NM001013101_fromGenomic.rn7
MOAP1_MusMusculus_BC055374_fromGenomic.mm39
MOAP1_CricetulusGriseus_XM027419169_fromGenomic.criGriChoV2
MOAP1_PteropusAlecto_XM006925542_fromGenomic.pteAle1
MOAP1_LoxodontaAfricana_XM023546521_fromGenomic.loxAfr3
MOAP1_FelisCatus_XM023255976_fromGenomic.felCat9
MOAP1_HomoSapiens_NM022151_fromGenomic.humT2T
MOAP1_MacacaMulatta_XM001089776_fromGenomic.rheMac10
MOAP1_CallithrixJacchus_XM009006472_fromGenomic.calJac4
MOAP1
iwasakiMePNMA-MS1_NotamacropusEugenii_fromGenomic.mMacEug1
kokosarM-PNMA_SarcophilusHarrisii_fromGenomic.sarHar1
iwasakiMdPNMA-MS1_MonodelphisDomestica_fromGenomic.mMonDom1
Marsupial PNMA
HMMhit_ChrysemysPictaBellii_JH586444_48
HMMhit_ChrysemysPictaBellii_JH586197_145
HMMhit_XenopusLaevis_NC_054382.1_129566
HMMhit_XenopusLaevis_NC_054376.1_71735
HMMhit_XenopusLaevis_NC_054371.1_172596
HMMhit_XenopusLaevis_NC_054387.1_46051
Outgroups (Metaviruses)
HMMhit_XenopusLaevis_NC_054379.1_55239
HMMhit_XenopusLaevis_NC_054371.1_94215
HMMhit_XenopusLaevis_NC_054371.1_19386
HMMhit_XenopusLaevis_NC_054381.1_157460
HMMhit_XenopusLaevis_NC_054372.1_204432
HMMhit_XenopusLaevis_NC_054371.1_12703
HMMhit_ChrysemysPictaBellii_JH584446_20318
HMMhit_ChrysemysPictaBellii_JH584890_888
HMMhit_AlligatorMississippiensis_JH733598_1427
HMMhit_AlligatorMississippiensis_JH733284_3313
HMMhit_AlligatorMississippiensis_JH733409_139
Gypsy-30_AMi_LTR_Gypsy
HMMhit_AlligatorMississippiensis_JH736845_80
100
100
95
30
49
100
100
95
100
59
83
100
56
84
73
91
100
23

Figure S4

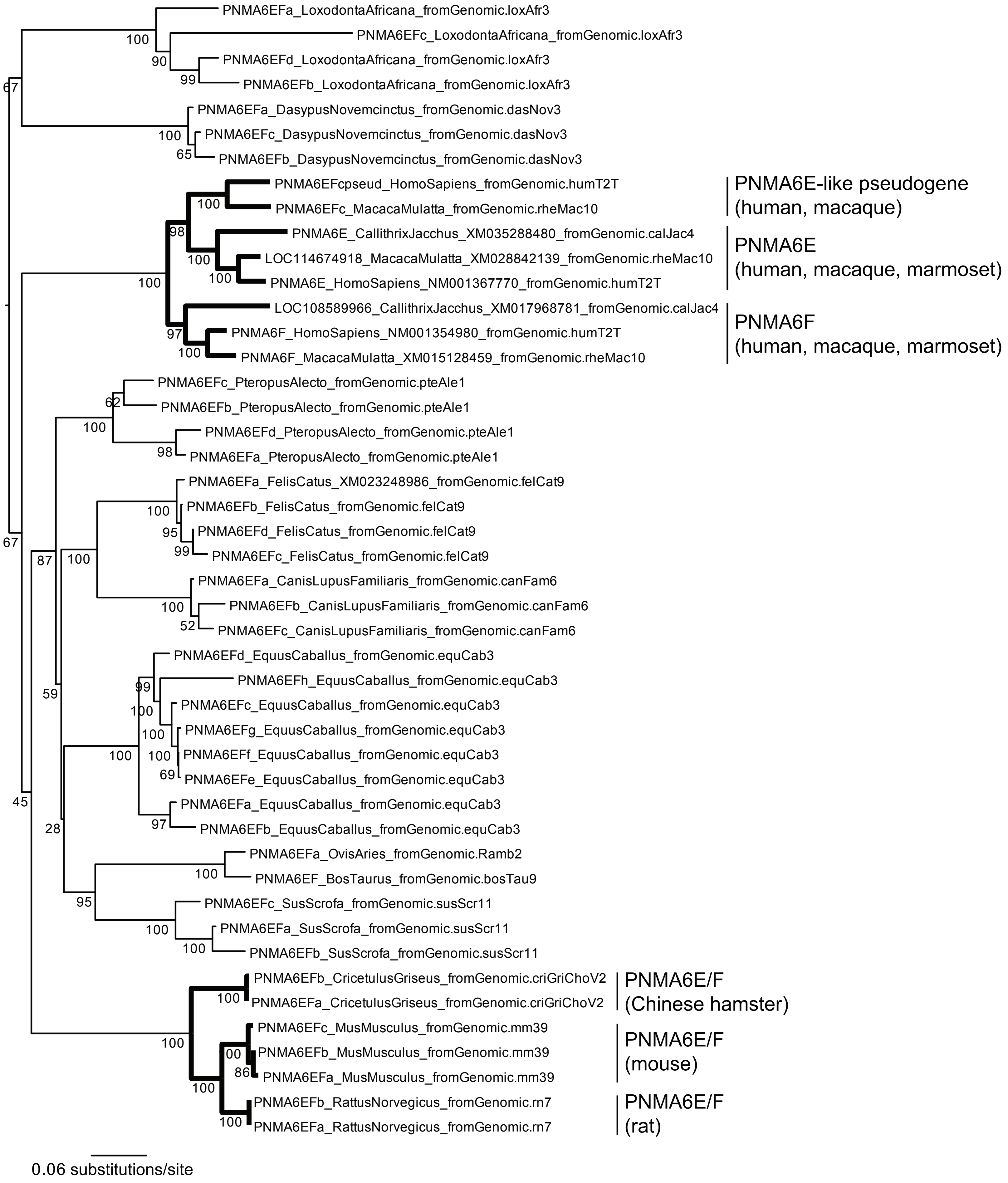
PNMA6EFa_LoxodontaAfricana_fromGenomic.loxAfr3
PNMA6EFc_LoxodontaAfricana_fromGenomic.loxAfr3
PNMA6EFd_LoxodontaAfricana_fromGenomic.loxAfr3
PNMA6EFb_LoxodontaAfricana_fromGenomic.loxAfr3
PNMA6EFa_DasypusNovemcinctus_fromGenomic.dasNov3
PNMA6EFc_DasypusNovemcinctus_fromGenomic.dasNov3
PNMA6EFb_DasypusNovemcinctus_fromGenomic.dasNov3
PNMA6EFcpseud_HomoSapiens_fromGenomic.humT2T
PNMA6EFc_MacacaMulatta_fromGenomic.rheMac10
PNMA6E_CallithrixJacchus_XM035288480_fromGenomic.calJac4
LOC114674918_MacacaMulatta_XM028842139_fromGenomic.rheMac10
PNMA6E_HomoSapiens_NM001367770_fromGenomic.humT2T
LOC108589966_CallithrixJacchus_XM017968781_fromGenomic.calJac4
PNMA6F_HomoSapiens_NM001354980_fromGenomic.humT2T
PNMA6F_MacacaMulatta_XM015128459_fromGenomic.rheMac10
PNMA6E-like pseudogene (human, macaque)
PNMA6E (human, macaque, marmoset)
PNMA6F (human, macaque, marmoset)
PNMA6EFc_PteropusAlecto_fromGenomic.pteAle1
PNMA6EFb_PteropusAlecto_fromGenomic.pteAle1
PNMA6EFd_PteropusAlecto_fromGenomic.pteAle1
PNMA6EFa_PteropusAlecto_fromGenomic.pteAle1
PNMA6EFa_FelisCatus_XM023248986_fromGenomic.felCat9
PNMA6EFb_FelisCatus_fromGenomic.felCat9
PNMA6EFd_FelisCatus_fromGenomic.felCat9
PNMA6EFc_FelisCatus_fromGenomic.felCat9
PNMA6EFa_CanisLupusFamiliaris_fromGenomic.canFam6
PNMA6EFb_CanisLupusFamiliaris_fromGenomic.canFam6
PNMA6EFc_CanisLupusFamiliaris_fromGenomic.canFam6
PNMA6EFd_EquusCaballus_fromGenomic.equCab3
PNMA6EFh_EquusCaballus_fromGenomic.equCab3
PNMA6EFc_EquusCaballus_fromGenomic.equCab3
PNMA6EFg_EquusCaballus_fromGenomic.equCab3
PNMA6EFf_EquusCaballus_fromGenomic.equCab3
PNMA6EFe_EquusCaballus_fromGenomic.equCab3
PNMA6EFa_EquusCaballus_fromGenomic.equCab3
PNMA6EFb_EquusCaballus_fromGenomic.equCab3
PNMA6EFa_OvisAries_fromGenomic.Ramb2
PNMA6EF_BosTaurus_fromGenomic.bosTau9
PNMA6EFc_SusScrofa_fromGenomic.susScr11
PNMA6EFa_SusScrofa_fromGenomic.susScr11
PNMA6EFb_SusScrofa_fromGenomic.susScr11
PNMA6EFb_CricetulusGriseus_fromGenomic.criGriChoV2
PNMA6EFa_CricetulusGriseus_fromGenomic.criGriChoV2
PNMA6E/F (Chinese hamster)
PNMA6EFc_MusMusculus_fromGenomic.mm39
PNMA6EFb_MusMusculus_fromGenomic.mm39
PNMA6EFa_MusMusculus_fromGenomic.mm39
PNMA6E/F (mouse)
PNMA6EFb_RattusNorvegicus_fromGenomic.rn7
PNMA6EFa_RattusNorvegicus_fromGenomic.rn7
PNMA6E/F (rat)
0.06 substitutions/site

Figure S5

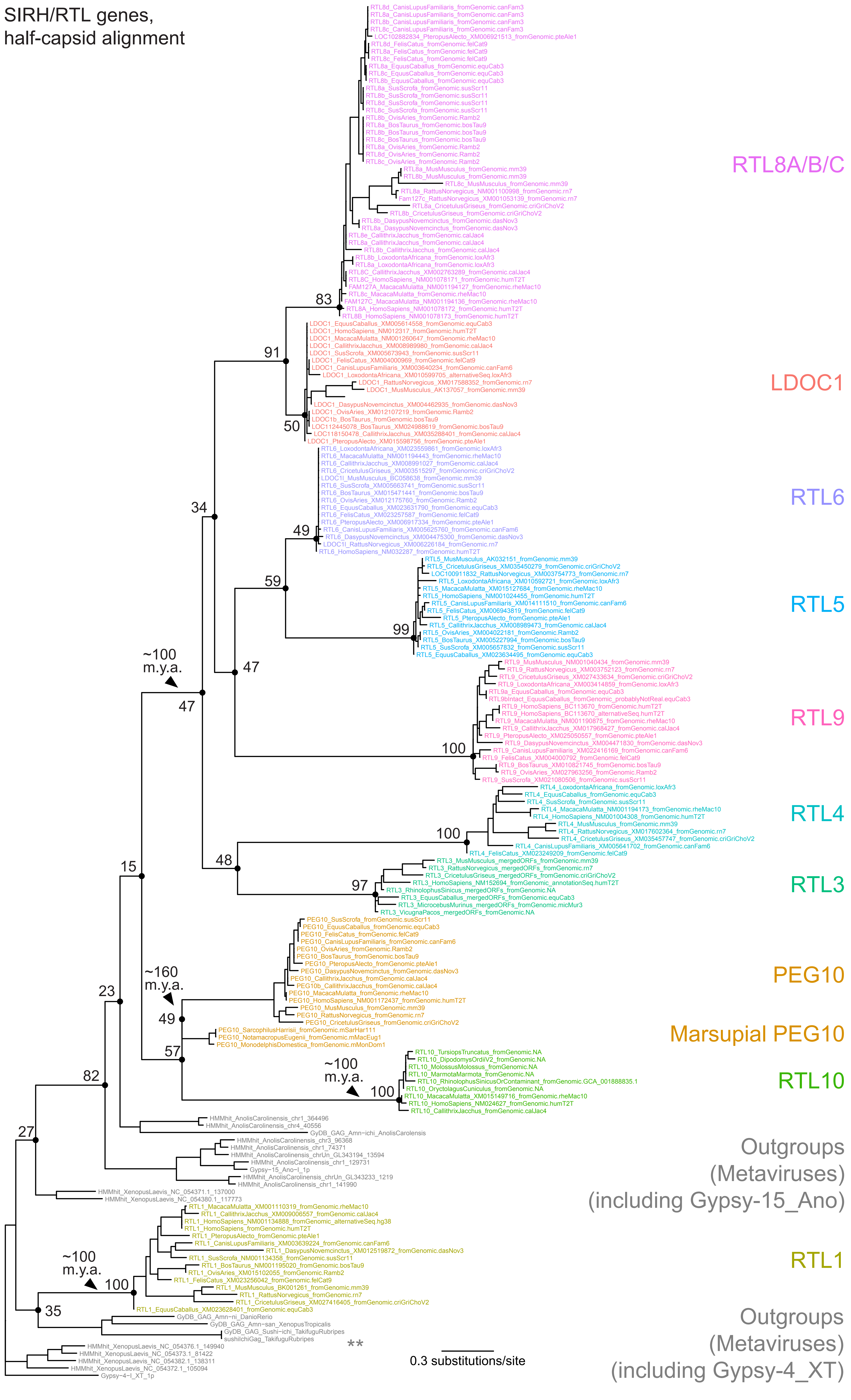

SIRH/RTL genes,
half-capsid alignment
RTL8d_CanisLupusFamiliaris_fromGenomic.canFam3
RTL8a_CanisLupusFamiliaris_fromGenomic.canFam3
RTL8b_CanisLupusFamiliaris_fromGenomic.canFam3
RTL8c_CanisLupusFamiliaris_fromGenomic.canFam3
LOC102882834_PteropusAlecto_XM006921513_fromGenomic.pteAle1
RTL8d_FelisCatus_fromGenomic.felCat9
RTL8a_FelisCatus_fromGenomic.felCat9
RTL8c_FelisCatus_fromGenomic.felCat9
RTL8a_EquusCaballus_fromGenomic.equCab3
RTL8c_EquusCaballus_fromGenomic.equCab3
RTL8b_EquusCaballus_fromGenomic.equCab3
RTL8a_SusScrofa_fromGenomic.susScr11
RTL8b_SusScrofa_fromGenomic.susScr11
RTL8d_SusScrofa_fromGenomic.susScr11
RTL8c_SusScrofa_fromGenomic.susScr11
RTL8b_OvisAries_fromGenomic.Ramb2
RTL8a_BosTaurus_fromGenomic.bosTau9
RTL8b_BosTaurus_fromGenomic.bosTau9
RTL8c_BosTaurus_fromGenomic.bosTau9
RTL8a_OvisAries_fromGenomic.Ramb2
RTL8d_OvisAries_fromGenomic.Ramb2
RTL8c_OvisAries_fromGenomic.Ramb2
RTL8a_MusMusculus_fromGenomic.mm39
RTL8b_MusMusculus_fromGenomic.mm39
RTL8c_MusMusculus_fromGenomic.mm39
RTL8a_RattusNorvegicus_NM001100998_fromGenomic.rn7
Fam127c_RattusNorvegicus_XM001053139_fromGenomic.rn7
RTL8a_CricetulusGriseus_fromGenomic.criGriChoV2
RTL8b_CricetulusGriseus_fromGenomic.criGriChoV2
RTL8b_DasypusNovemcinctus_fromGenomic.dasNov3
RTL8a_DasypusNovemcinctus_fromGenomic.dasNov3
RTL8e_CallithrixJacchus_fromGenomic.calJac4
RTL8a_CallithrixJacchus_fromGenomic.calJac4
RTL8b_CallithrixJacchus_fromGenomic.calJac4
RTL8b_LoxodontaAfricana_fromGenomic.loxAfr3
RTL8a_LoxodontaAfricana_fromGenomic.loxAfr3
RTL8C_CallithrixJacchus_XM002763289_fromGenomic.calJac4
RTL8C_HomoSapiens_NM001078171_fromGenomic.humT2T
FAM127A_MacacaMulatta_NM001194127_fromGenomic.rheMac10
RTL8c_MacacaMulatta_fromGenomic.rheMac10
FAM127C_MacacaMulatta_NM001194136_fromGenomic.rheMac10
RTL8A_HomoSapiens_NM001078172_fromGenomic.humT2T
RTL8B_HomoSapiens_NM001078173_fromGenomic.humT2T
RTL8A/B/C
83
91
LDOC1_EquusCaballus_XM005614558_fromGenomic.equCab3
LDOC1_HomoSapiens_NM012317_fromGenomic.humT2T
LDOC1_MacacaMulatta_NM001260647_fromGenomic.rheMac10
LDOC1_CallithrixJacchus_XM008989980_fromGenomic.calJac4
LDOC1_SusScrofa_XM005673943_fromGenomic.susScr11
LDOC1_FelisCatus_XM004000969_fromGenomic.felCat9
LDOC1_CanisLupusFamiliaris_XM003640234_fromGenomic.canFam6
LDOC1_LoxodontaAfricana_XM010599705_alternativeSeq.loxAfr3
LDOC1_RattusNorvegicus_XM017588352_fromGenomic.rn7
LDOC1_MusMusculus_AK137057_fromGenomic.mm39
LDOC1_DasypusNovemcinctus_XM004462935_fromGenomic.dasNov3
LDOC1_OvisAries_XM012107219_fromGenomic.Ramb2
LDOC1b_BosTaurus_fromGenomic.bosTau9
LOC112445078_BosTaurus_XM024988619_fromGenomic.bosTau9
LOC118150478_CallithrixJacchus_XM035288401_fromGenomic.calJac4
LDOC1_PteropusAlecto_XM015598756_fromGenomic.pteAle1
50
LDOC1
RTL6_LoxodontaAfricana_XM023559861_fromGenomic.loxAfr3
RTL6_MacacaMulatta_NM001194443_fromGenomic.rheMac10
RTL6_CallithrixJacchus_XM008991027_fromGenomic.calJac4
RTL6_CricetulusGriseus_XM003515297_fromGenomic.criGriChoV2
LDOC1l_MusMusculus_BC058638_fromGenomic.mm39
RTL6_SusScrofa_XM005663741_fromGenomic.susScr11
RTL6_BosTaurus_XM015471441_fromGenomic.bosTau9
RTL6_OvisAries_XM012175760_fromGenomic.Ramb2
RTL6_EquusCaballus_XM023631790_fromGenomic.equCab3
RTL6_FelisCatus_XM023257587_fromGenomic.felCat9
RTL6_PteropusAlecto_XM006917334_fromGenomic.pteAle1
RTL6_CanisLupusFamiliaris_XM005625760_fromGenomic.canFam6
RTL6_DasypusNovemcinctus_XM004475300_fromGenomic.dasNov3
LDOC1l_RattusNorvegicus_XM006226184_fromGenomic.rn7
RTL6_HomoSapiens_NM032287_fromGenomic.humT2T
RTL6
34
49
59
RTL5_MusMusculus_AK032151_fromGenomic.mm39
RTL5_CricetulusGriseus_XM035450279_fromGenomic.criGriChoV2
LOC100911832_RattusNorvegicus_XM003754773_fromGenomic.rn7
RTL5_LoxodontaAfricana_XM010592721_fromGenomic.loxAfr3
RTL5_MacacaMulatta_XM015127684_fromGenomic.rheMac10
RTL5_HomoSapiens_NM001024455_fromGenomic.humT2T
RTL5_CanisLupusFamiliaris_XM014111510_fromGenomic.canFam6
RTL5_FelisCatus_XM006943819_fromGenomic.felCat9
RTL5_PteropusAlecto_fromGenomic.pteAle1
RTL5_CallithrixJacchus_XM008989473_fromGenomic.calJac4
RTL5_OvisAries_XM004022181_fromGenomic.Ramb2
RTL5_BosTaurus_XM005227994_fromGenomic.bosTau9
RTL5_SusScrofa_XM005657832_fromGenomic.susScr11
RTL5_EquusCaballus_XM023634495_fromGenomic.equCab3
99
RTL5
~100
m.y.a.
47
47
RTL9_MusMusculus_NM001040434_fromGenomic.mm39
RTL9_RattusNorvegicus_XM003752123_fromGenomic.rn7
RTL9_CricetulusGriseus_XM027433634_fromGenomic.criGriChoV2
RTL9_LoxodontaAfricana_XM003414859_fromGenomic.loxAfr3
RTL9a_EquusCaballus_fromGenomic.equCab3
RTL9bIntact_EquusCaballus_fromGenomic_probablyNotReal.equCab3
RTL9_HomoSapiens_BC113670_fromGenomic.humT2T
RTL9_HomoSapiens_BC113670_alternativeSeq.humT2T
RTL9_MacacaMulatta_NM001190875_fromGenomic.rheMac10
RTL9_CallithrixJacchus_XM017968427_fromGenomic.calJac4
RTL9_PteropusAlecto_XM025050557_fromGenomic.pteAle1
RTL9_DasypusNovemcinctus_XM004471830_fromGenomic.dasNov3
RTL9_CanisLupusFamiliaris_XM022416169_fromGenomic.canFam6
RTL9_FelisCatus_XM004000792_fromGenomic.felCat9
RTL9_BosTaurus_XM010821745_fromGenomic.bosTau9
RTL9_OvisAries_XM027963256_fromGenomic.Ramb2
RTL9_SusScrofa_XM021080506_fromGenomic.susScr11
100
RTL9
RTL4_LoxodontaAfricana_fromGenomic.loxAfr3
RTL4_EquusCaballus_fromGenomic.equCab3
RTL4_SusScrofa_fromGenomic.susScr11
RTL4_MacacaMulatta_NM001194173_fromGenomic.rheMac10
RTL4_HomoSapiens_NM001004308_fromGenomic.humT2T
RTL4_MusMusculus_fromGenomic.mm39
RTL4_RattusNorvegicus_XM017602364_fromGenomic.rn7
RTL4_CricetulusGriseus_XM035457747_fromGenomic.criGriChoV2
RTL4_CanisLupusFamiliaris_XM005641702_fromGenomic.canFam6
RTL4_FelisCatus_XM023249209_fromGenomic.felCat9
100
RTL4
48
RTL3_MusMusculus_mergedORFs_fromGenomic.mm39
RTL3_RattusNorvegicus_mergedORFs_fromGenomic.rn7
RTL3_CricetulusGriseus_mergedORFs_fromGenomic.criGriChoV2
RTL3_HomoSapiens_NM152694_fromGenomic_annotationSeq.humT2T
RTL3_RhinolophusSinicus_mergedORFs_fromGenomic.NA
RTL3_EquusCaballus_mergedORFs_fromGenomic.equCab3
RTL3_MicrocebusMurinus_mergedORFs_fromGenomic.micMur3
RTL3_VicugnaPacos_mergedORFs_fromGenomic.NA
97
RTL3
15
PEG10_SusScrofa_fromGenomic.susScr11
PEG10_EquusCaballus_fromGenomic.equCab3
PEG10_FelisCatus_fromGenomic.felCat9
PEG10_CanisLupusFamiliaris_fromGenomic.canFam6
PEG10_OvisAries_fromGenomic.Ramb2
PEG10_BosTaurus_fromGenomic.bosTau9
PEG10_PteropusAlecto_fromGenomic.pteAle1
PEG10_DasypusNovemcinctus_fromGenomic.dasNov3
PEG10_CallithrixJacchus_fromGenomic.calJac4
PEG10b_CallithrixJacchus_fromGenomic.calJac4
PEG10_MacacaMulatta_fromGenomic.rheMac10
PEG10_HomoSapiens_NM001172437_fromGenomic.humT2T
PEG10_MusMusculus_fromGenomic.mm39
PEG10_RattusNorvegicus_fromGenomic.rn7
PEG10_CricetulusGriseus_fromGenomic.criGriChoV2
PEG10
~160
m.y.a.
23
49
PEG10_SarcophilusHarrisii_fromGenomic.mSarHar111
PEG10_NotamacropusEugenii_fromGenomic.mMacEug1
PEG10_MonodelphisDomestica_fromGenomic.mMonDom1
Marsupial PEG10
57
RTL10_TursiopsTruncatus_fromGenomic.NA
RTL10_DipodomysOrdiiV2_fromGenomic.NA
RTL10_MolossusMolossus_fromGenomic.NA
RTL10_MarmotaMarmota_fromGenomic.NA
RTL10_RhinolophusSinicusOrContaminant_fromGenomic.GCA_001888835.1
RTL10_OryctolagusCuniculus_fromGenomic.NA
RTL10_MacacaMulatta_XM015149716_fromGenomic.rheMac10
RTL10_HomoSapiens_NM024627_fromGenomic.humT2T
RTL10_CallithrixJacchus_fromGenomic.calJac4
~100
m.y.a.
100
RTL10
82
HMMhit_AnolisCarolinensis_chr1_364496
HMMhit_AnolisCarolinensis_chr4_40556
GyDB_GAG_Amn-ichi_AnolisCarolensis
27
HMMhit_AnolisCarolinensis_chr3_96368
HMMhit_AnolisCarolinensis_chr1_74371
HMMhit_AnolisCarolinensis_chrUn_GL343194_13594
HMMhit_AnolisCarolinensis_chr1_129731
Gypsy-15_Ano-I_1p
HMMhit_AnolisCarolinensis_chrUn_GL343233_1219
HMMhit_AnolisCarolinensis_chr1_141990
HMMhit_XenopusLaevis_NC_054371.1_137000
HMMhit_XenopusLaevis_NC_054380.1_117773
Outgroups
(Metaviruses)
(including Gypsy-15_Ano)
RTL1_MacacaMulatta_XM001110319_fromGenomic.rheMac10
RTL1_CallithrixJacchus_XM009006557_fromGenomic.calJac4
RTL1_HomoSapiens_NM001134888_fromGenomic_alternativeSeq.hg38
RTL1_HomoSapiens_fromGenomic.humT2T
RTL1_PteropusAlecto_fromGenomic.pteAle1
RTL1_CanisLupusFamiliaris_XM003639224_fromGenomic.canFam6
RTL1_DasypusNovemcinctus_XM012519872_fromGenomic.dasNov3
RTL1_SusScrofa_NM001134358_fromGenomic.susScr11
RTL1_BosTaurus_NM001195020_fromGenomic.bosTau9
RTL1_OvisAries_XM015102055_fromGenomic.Ramb2
RTL1_FelisCatus_XM023256042_fromGenomic.felCat9
RTL1_MusMusculus_BK001261_fromGenomic.mm39
RTL1_RattusNorvegicus_fromGenomic.rn7
RTL1_CricetulusGriseus_XM027416405_fromGenomic.criGriChoV2
RTL1_EquusCaballus_XM023628401_fromGenomic.equCab3
~100
m.y.a.
100
RTL1
35
GyDB_GAG_Amn-ni_DanioRerio
GyDB_GAG_Amn-san_XenopusTropicalis
GyDB_GAG_Sushi-ichi_TakifuguRubripes
sushiIchiGag_TakifuguRubripes
**
HMMhit_XenopusLaevis_NC_054376.1_149940
HMMhit_XenopusLaevis_NC_054373.1_81422
HMMhit_XenopusLaevis_NC_054382.1_138311
HMMhit_XenopusLaevis_NC_054372.1_105094
Gypsy-4-I_XT_1p
0.3 substitutions/site
Outgroups
(Metaviruses)
(including Gypsy-4_XT)

Figure S6

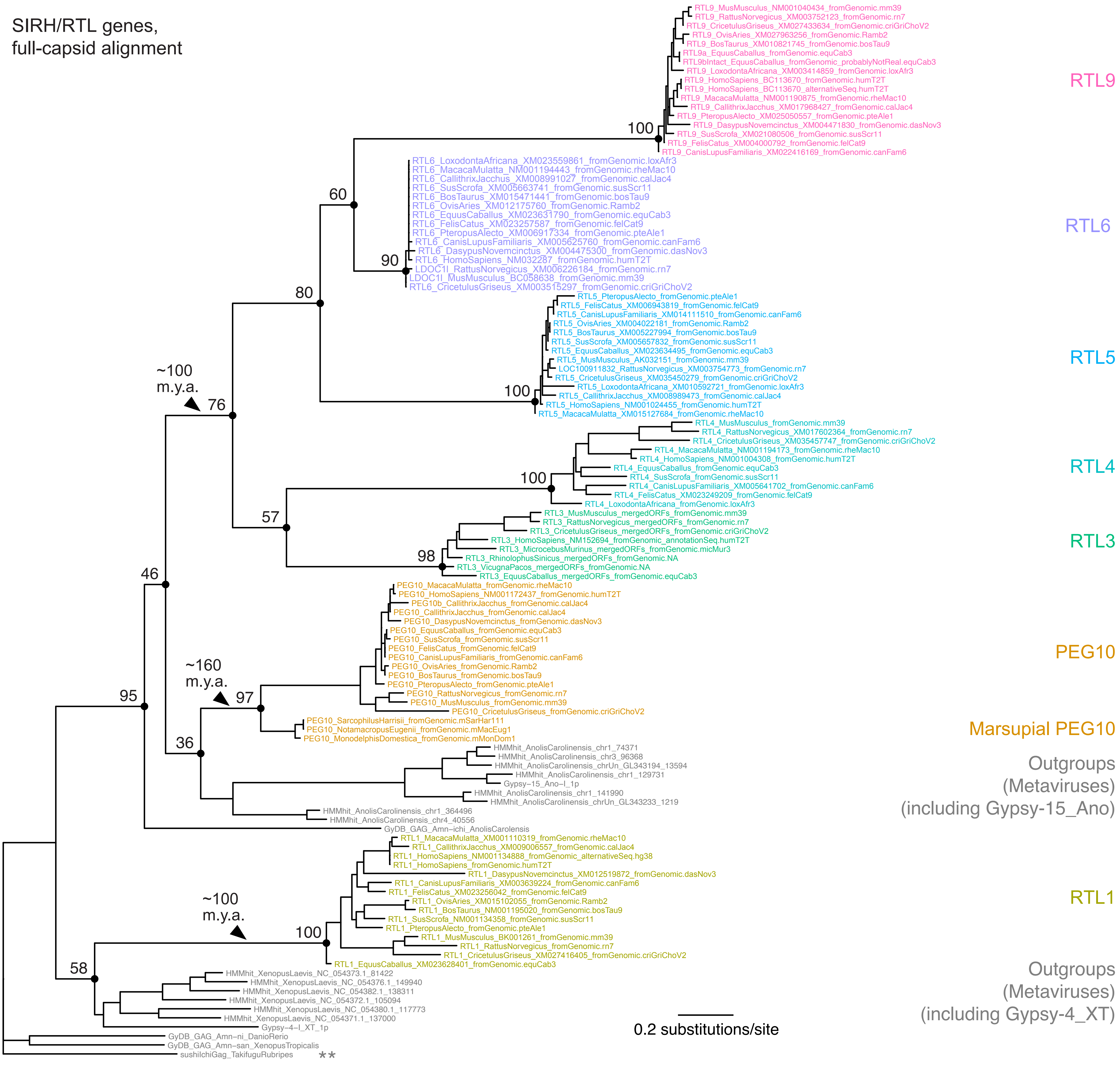

SIRH/RTL genes,
full-capsid alignment
RTL9_MusMusculus_NM001040434_fromGenomic.mm39
RTL9_RattusNorvegicus_XM003752123_fromGenomic.rn7
RTL9_CricetulusGriseus_XM027433634_fromGenomic.criGriChoV2
RTL9_OvisAries_XM027963256_fromGenomic.Ramb2
RTL9_BosTaurus_XM010821745_fromGenomic.bosTau9
RTL9a_EquusCaballus_fromGenomic.equCab3
RTL9bIntact_EquusCaballus_fromGenomic_probablyNotReal.equCab3
RTL9_LoxodontaAfricana_XM003414859_fromGenomic.loxAfr3
RTL9_HomoSapiens_BC113670_fromGenomic.humT2T
RTL9_HomoSapiens_BC113670_alternativeSeq.humT2T
RTL9_MacacaMulatta_NM001190875_fromGenomic.rheMac10
RTL9_CallithrixJacchus_XM017968427_fromGenomic.calJac4
RTL9_PteropusAlecto_XM025050557_fromGenomic.pteAle1
RTL9_DasypusNovemcinctus_XM004471830_fromGenomic.dasNov3
RTL9_SusScrofa_XM021080506_fromGenomic.susScr11
RTL9_FelisCatus_XM004000792_fromGenomic.felCat9
RTL9_CanisLupusFamiliaris_XM022416169_fromGenomic.canFam6
RTL9
100
RTL6_LoxodontaAfricana_XM023559861_fromGenomic.loxAfr3
RTL6_MacacaMulatta_NM001194443_fromGenomic.rheMac10
RTL6_CallithrixJacchus_XM008991027_fromGenomic.calJac4
RTL6_SusScrofa_XM005663741_fromGenomic.susScr11
RTL6_BosTaurus_XM015471441_fromGenomic.bosTau9
RTL6_OvisAries_XM012175760_fromGenomic.Ramb2
RTL6_EquusCaballus_XM023631790_fromGenomic.equCab3
RTL6_FelisCatus_XM023257587_fromGenomic.felCat9
RTL6_PteropusAlecto_XM006917334_fromGenomic.pteAle1
RTL6_CanisLupusFamiliaris_XM005625760_fromGenomic.canFam6
RTL6_DasypusNovemcinctus_XM004475300_fromGenomic.dasNov3
RTL6_HomoSapiens_NM032287_fromGenomic.humT2T
LDOC1l_RattusNorvegicus_XM006226184_fromGenomic.rn7
LDOC1l_MusMusculus_BC058638_fromGenomic.mm39
RTL6_CricetulusGriseus_XM003515297_fromGenomic.criGriChoV2
RTL6
60
90
80
RTL5_PteropusAlecto_fromGenomic.pteAle1
RTL5_FelisCatus_XM006943819_fromGenomic.felCat9
RTL5_CanisLupusFamiliaris_XM014111510_fromGenomic.canFam6
RTL5_OvisAries_XM004022181_fromGenomic.Ramb2
RTL5_BosTaurus_XM005227994_fromGenomic.bosTau9
RTL5_SusScrofa_XM005657832_fromGenomic.susScr11
RTL5_EquusCaballus_XM023634495_fromGenomic.equCab3
RTL5_MusMusculus_AK032151_fromGenomic.mm39
LOC100911832_RattusNorvegicus_XM003754773_fromGenomic.rn7
RTL5_CricetulusGriseus_XM035450279_fromGenomic.criGriChoV2
RTL5_LoxodontaAfricana_XM010592721_fromGenomic.loxAfr3
RTL5_CallithrixJacchus_XM008989473_fromGenomic.calJac4
RTL5_HomoSapiens_NM001024455_fromGenomic.humT2T
RTL5_MacacaMulatta_XM015127684_fromGenomic.rheMac10
RTL5
100
~100
m.y.a.
76
RTL4_MusMusculus_fromGenomic.mm39
RTL4_RattusNorvegicus_XM017602364_fromGenomic.rn7
RTL4_CricetulusGriseus_XM035457747_fromGenomic.criGriChoV2
RTL4_MacacaMulatta_NM001194173_fromGenomic.rheMac10
RTL4_HomoSapiens_NM001004308_fromGenomic.humT2T
RTL4_EquusCaballus_fromGenomic.equCab3
RTL4_SusScrofa_fromGenomic.susScr11
RTL4_CanisLupusFamiliaris_XM005641702_fromGenomic.canFam6
RTL4_FelisCatus_XM023249209_fromGenomic.felCat9
RTL4_LoxodontaAfricana_fromGenomic.loxAfr3
RTL4
100
57
RTL3_MusMusculus_mergedORFs_fromGenomic.mm39
RTL3_RattusNorvegicus_mergedORFs_fromGenomic.rn7
RTL3_CricetulusGriseus_mergedORFs_fromGenomic.criGriChoV2
RTL3_HomoSapiens_NM152694_fromGenomic_annotationSeq.humT2T
RTL3_MicrocebusMurinus_mergedORFs_fromGenomic.micMur3
RTL3_RhinolophusSinicus_mergedORFs_fromGenomic.NA
RTL3_VicugnaPacos_mergedORFs_fromGenomic.NA
RTL3_EquusCaballus_mergedORFs_fromGenomic.equCab3
RTL3
98
46
PEG10_MacacaMulatta_fromGenomic.rheMac10
PEG10_HomoSapiens_NM001172437_fromGenomic.humT2T
PEG10b_CallithrixJacchus_fromGenomic.calJac4
PEG10_CallithrixJacchus_fromGenomic.calJac4
PEG10_DasypusNovemcinctus_fromGenomic.dasNov3
PEG10_EquusCaballus_fromGenomic.equCab3
PEG10_SusScrofa_fromGenomic.susScr11
PEG10_FelisCatus_fromGenomic.felCat9
PEG10_CanisLupusFamiliaris_fromGenomic.canFam6
PEG10_OvisAries_fromGenomic.Ramb2
PEG10_BosTaurus_fromGenomic.bosTau9
PEG10_PteropusAlecto_fromGenomic.pteAle1
PEG10_RattusNorvegicus_fromGenomic.rn7
PEG10_MusMusculus_fromGenomic.mm39
PEG10_CricetulusGriseus_fromGenomic.criGriChoV2
PEG10
~160
m.y.a.
97
PEG10_SarcophilusHarrisii_fromGenomic.mSarHar111
PEG10_NotamacropusEugenii_fromGenomic.mMacEug1
PEG10_MonodelphisDomestica_fromGenomic.mMonDom1
Marsupial PEG10
95
36
HMMhit_AnolisCarolinensis_chr1_74371
HMMhit_AnolisCarolinensis_chr3_96368
HMMhit_AnolisCarolinensis_chrUn_GL343194_13594
HMMhit_AnolisCarolinensis_chr1_129731
Gypsy−15_Ano−I_1p
HMMhit_AnolisCarolinensis_chr1_141990
HMMhit_AnolisCarolinensis_chrUn_GL343233_1219
HMMhit_AnolisCarolinensis_chr1_364496
HMMhit_AnolisCarolinensis_chr4_40556
GyDB_GAG_Amn−ichi_AnolisCarolensis
Outgroups
(Metaviruses)
(including Gypsy-15_Ano)
RTL1_MacacaMulatta_XM001110319_fromGenomic.rheMac10
RTL1_CallithrixJacchus_XM009006557_fromGenomic.calJac4
RTL1_HomoSapiens_NM001134888_fromGenomic_alternativeSeq.hg38
RTL1_HomoSapiens_fromGenomic.humT2T
RTL1_DasypusNovemcinctus_XM012519872_fromGenomic.dasNov3
RTL1_CanisLupusFamiliaris_XM003639224_fromGenomic.canFam6
RTL1_FelisCatus_XM023256042_fromGenomic.felCat9
RTL1_OvisAries_XM015102055_fromGenomic.Ramb2
RTL1_BosTaurus_NM001195020_fromGenomic.bosTau9
RTL1_SusScrofa_NM001134358_fromGenomic.susScr11
RTL1_PteropusAlecto_fromGenomic.pteAle1
RTL1_MusMusculus_BK001261_fromGenomic.mm39
RTL1_RattusNorvegicus_fromGenomic.rn7
RTL1_CricetulusGriseus_XM027416405_fromGenomic.criGriChoV2
RTL1_EquusCaballus_XM023628401_fromGenomic.equCab3
RTL1
~100
m.y.a.
100
58
HMMhit_XenopusLaevis_NC_054373.1_81422
HMMhit_XenopusLaevis_NC_054376.1_149940
HMMhit_XenopusLaevis_NC_054382.1_138311
HMMhit_XenopusLaevis_NC_054372.1_105094
HMMhit_XenopusLaevis_NC_054380.1_117773
HMMhit_XenopusLaevis_NC_054371.1_137000
Gypsy−4−I_XT_1p
GyDB_GAG_Amn−ni_DanioRerio
GyDB_GAG_Amn−san_XenopusTropicalis
sushiIchiGag_TakifuguRubripes
**
Outgroups
(Metaviruses)
(including Gypsy-4_XT)
0.2 substitutions/site

Figure S7

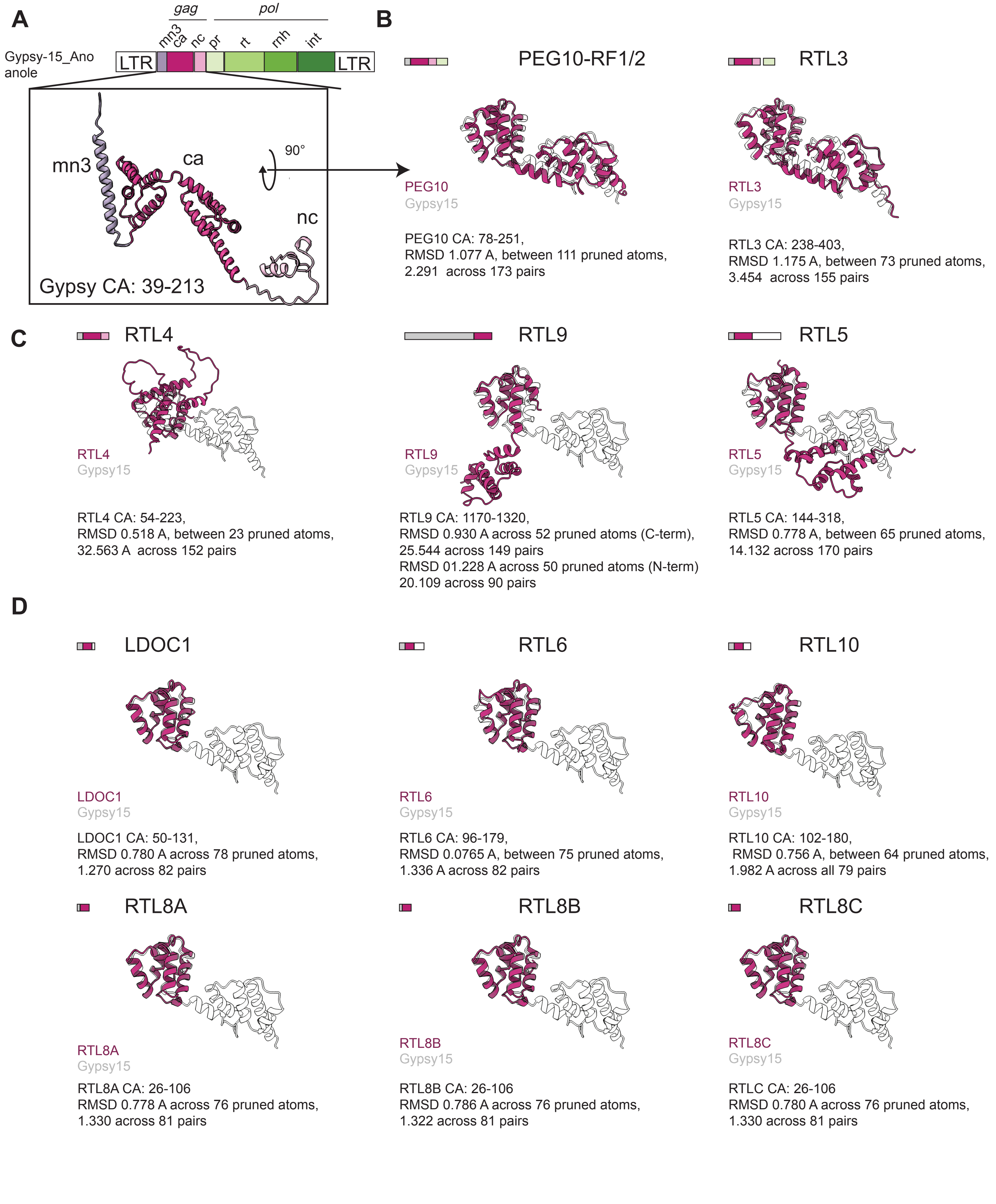
A
gag
pol
Gypsy-15_Ano anole
LTR
mn3
ca
nc
pr
rt
rnh
int
LTR
mn3
ca
90°
nc
Gypsy CA: 39-213
B
PEG10-RF1/2
PEG10
Gypsy15
PEG10 CA: 78-251,
RMSD 1.077 A, between 111 pruned atoms,
2.291 across 173 pairs
RTL3
RTL3
Gypsy15
RTL3 CA: 238-403,
RMSD 1.175 A, between 73 pruned atoms,
3.454 across 155 pairs
C
RTL4
RTL4
Gypsy15
RTL4 CA: 54-223,
RMSD 0.518 A, between 23 pruned atoms,
32.563 A across 152 pairs
RTL9
RTL9
Gypsy15
RTL9 CA: 1170-1320,
RMSD 0.930 A across 52 pruned atoms (C-term),
25.544 across 149 pairs
RMSD 01.228 A across 50 pruned atoms (N-term)
20.109 across 90 pairs
RTL5
RTL5
Gypsy15
RTL5 CA: 144-318,
RMSD 0.778 A, between 65 pruned atoms,
14.132 across 170 pairs
D
LDOC1
LDOC1
Gypsy15
LDOC1 CA: 50-131,
RMSD 0.780 A across 78 pruned atoms,
1.270 across 82 pairs
RTL6
RTL6
Gypsy15
RTL6 CA: 96-179,
RMSD 0.0765 A, between 75 pruned atoms,
1.336 A across 82 pairs
RTL10
RTL10
Gypsy15
RTL10 CA: 102-180,
RMSD 0.756 A, between 64 pruned atoms,
1.982 A across all 79 pairs
RTL8A
RTL8A
Gypsy15
RTL8A CA: 26-106
RMSD 0.778 A across 76 pruned atoms,
1.330 across 81 pairs
RTL8B
RTL8B
Gypsy15
RTL8B CA: 26-106
RMSD 0.786 A across 76 pruned atoms,
1.322 across 81 pairs
RTL8C
RTL8C
Gypsy15
RTLC CA: 26-106
RMSD 0.780 A across 76 pruned atoms,
1.330 across 81 pairs

Figure S8

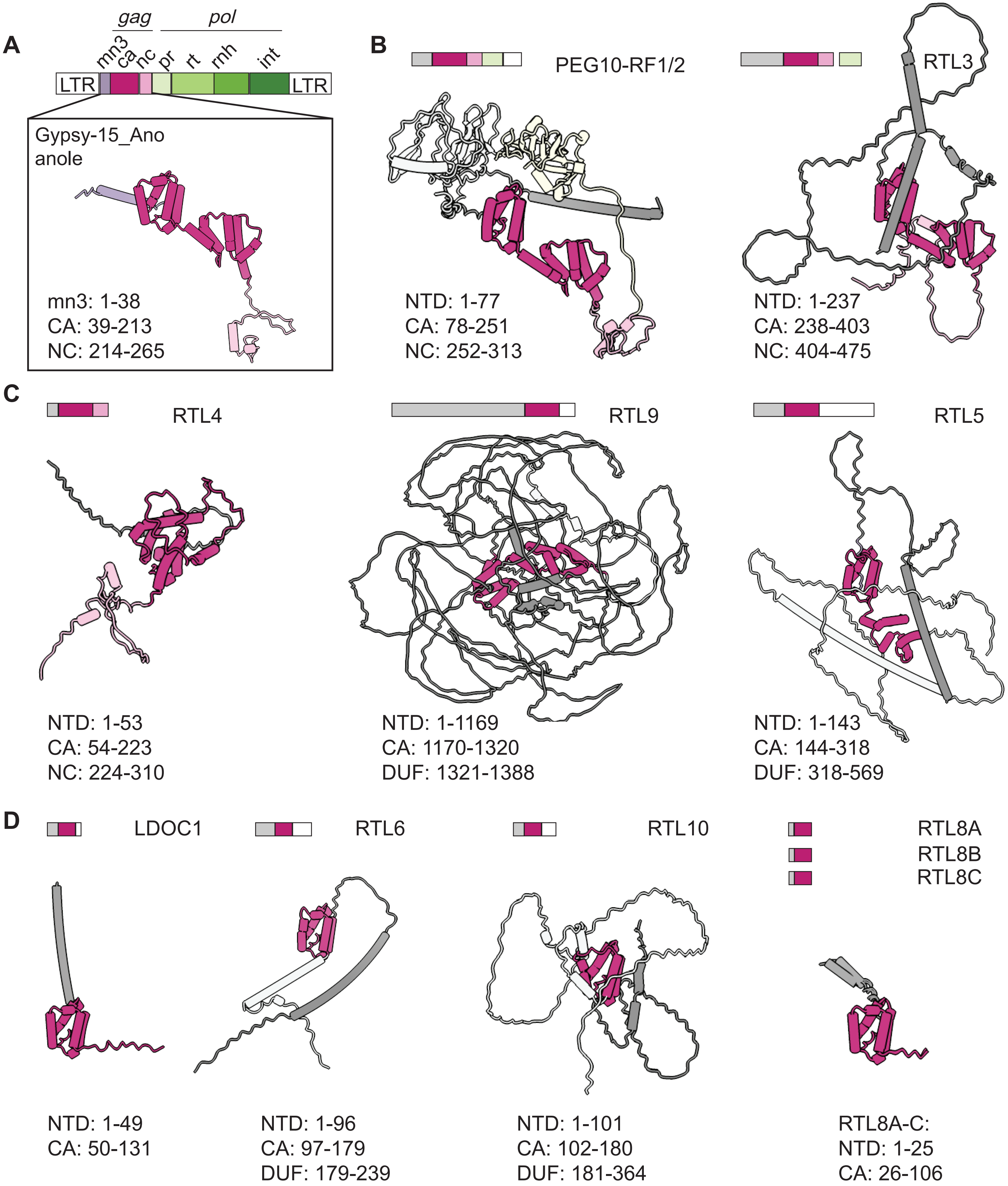
A
gag
pol
mn3
ca
nc
pr
rt
rnh
int
LTR
LTR
Gypsy-15_Ano
anole
mn3: 1-38
CA: 39-213
NC: 214-265
B
PEG10-RF1/2
NTD: 1-77
CA: 78-251
NC: 252-313
RTL3
NTD: 1-237
CA: 238-403
NC: 404-475
C
RTL4
NTD: 1-53
CA: 54-223
NC: 224-310
RTL9
NTD: 1-1169
CA: 1170-1320
DUF: 1321-1388
RTL5
NTD: 1-143
CA: 144-318
DUF: 318-569
D
LDOC1
NTD: 1-49
CA: 50-131
RTL6
NTD: 1-96
CA: 97-179
DUF: 179-239
RTL10
NTD: 1-101
CA: 102-180
DUF: 181-364
RTL8A
RTL8B
RTL8C
RTL8A-C:
NTD: 1-25
CA: 26-106

Figure S9

A

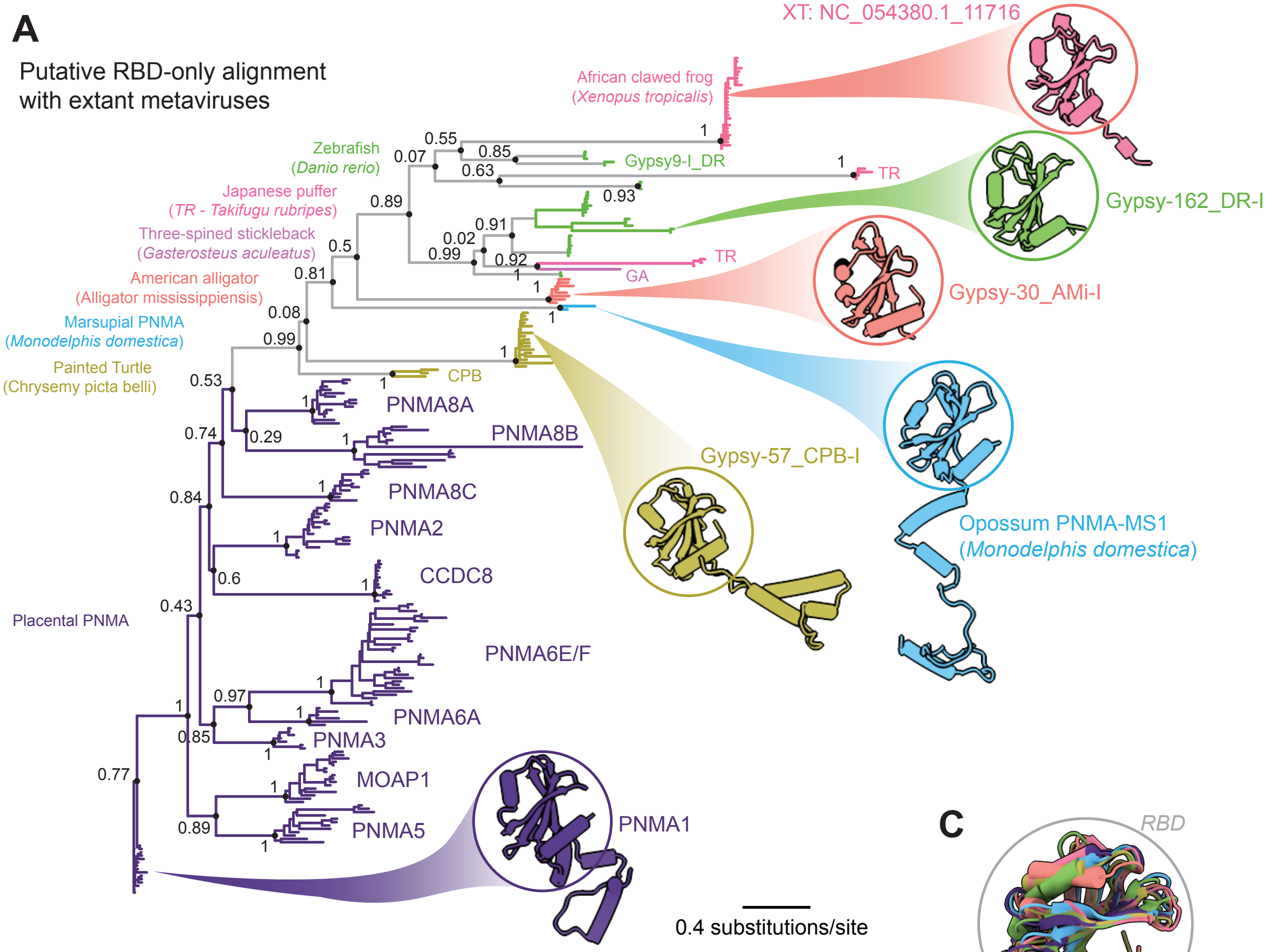

B

RBD	mmaker to	num pruned pairs	pruned RMSD	num pairs	all pairs RMSD
Gypsy-30_AMi-I	PNMA1	61	0.985	96	2.554
Gypsy-57_CPB-I	PNMA1	60	0.954	97	3.502
XT: NC_054380.1_11716	PNMA1	62	1.1	98	5.826
Gypsy-162_DR-I	PNMA1	49	0.93	95	4.185
PNMA-MS1 (opossum)	PNMA1	64	1.03	99	2.448

C

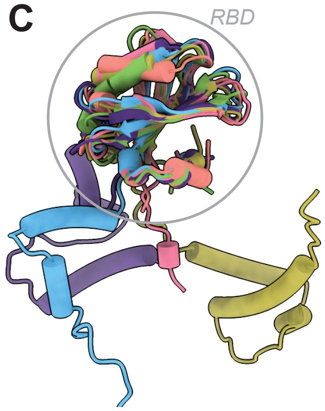

Figure S10

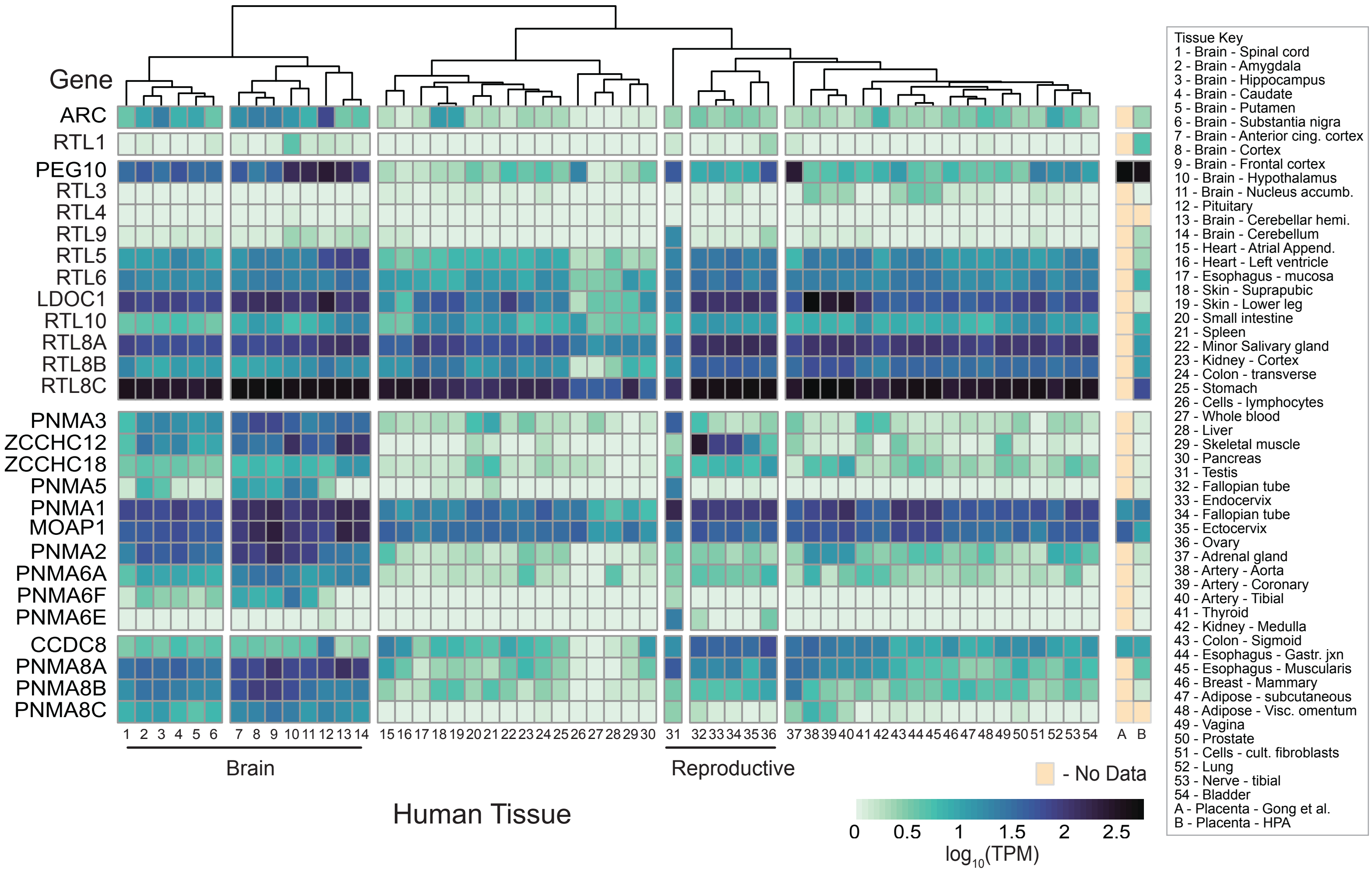
Gene
ARC
RTL1
PEG10
RTL3
RTL4
RTL9
RTL5
RTL6
LDOC1
RTL10
RTL8A
RTL8B
RTL8C
PNMA3
ZCCHC12
ZCCHC18
PNMA5
PNMA1
MOAP1
PNMA2
PNMA6A
PNMA6F
PNMA6E
CCDC8
PNMA8A
PNMA8B
PNMA8C
1 2 3 4 5 6 7 8 9 10 11 12 13 14 15 16 17 18 19 20 21 22 23 24 25 26 27 28 29 30 31 32 33 34 35 36 37 38 39 40 41 42 43 44 45 46 47 48 49 50 51 52 53 54 A B
Brain
Reproductive
Human Tissue
- No Data
0 0.5 1 1.5 2 2.5
log10(TPM)
Tissue Key
1 - Brain - Spinal cord
2 - Brain - Amygdala
3 - Brain - Hippocampus
4 - Brain - Caudate
5 - Brain - Putamen
6 - Brain - Substantia nigra
7 - Brain - Anterior cing. cortex
8 - Brain - Cortex
9 - Brain - Frontal cortex
10 - Brain - Hypothalamus
11 - Brain - Nucleus accumb.
12 - Pituitary
13 - Brain - Cerebellar hemi.
14 - Brain - Cerebellum
15 - Heart - Atrial Append.
16 - Heart - Left ventricle
17 - Esophagus - mucosa
18 - Skin - Suprapubic
19 - Skin - Lower leg
20 - Small intestine
21 - Spleen
22 - Minor Salivary gland
23 - Kidney - Cortex
24 - Colon - transverse
25 - Stomach
26 - Cells - lymphocytes
27 - Whole blood
28 - Liver
29 - Skeletal muscle
30 - Pancreas
31 - Testis
32 - Fallopian tube
33 - Endocervix
34 - Fallopian tube
35 - Ectocervix
36 - Ovary
37 - Adrenal gland
38 - Artery - Aorta
39 - Artery - Coronary
40 - Artery - Tibial
41 - Thyroid
42 - Kidney - Medulla
43 - Colon - Sigmoid
44 - Esophagus - Gastr. jxn
45 - Esophagus - Muscularis
46 - Breast - Mammary
47 - Adipose - subcutaneous
48 - Adipose - Visc. omentum
49 - Vagina
50 - Prostate
51 - Cells - cult. fibroblasts
52 - Lung
53 - Nerve - tibial
54 - Bladder
A - Placenta - Gong et al.
B - Placenta - HPA

Figure S11
