# Supplementary material for: The Diverse Evolutionary Histories of Domesticated Metaviral Capsid Genes in Mammals
Source: Mol Biol Evol. 2024 Mar 20;41(4):msae061. doi: 10.1093/molbev/msae061 (PMC11011659; doi:10.1093/molbev/msae061)
Supplement: msae061_Supplementary_Data [file msae061_supplementary_data.zip › TableS1_HMMstatistics_v2.pdf]

Table S1. Model statistics for custom capsid Hidden Markov Models (HMMs) build from multiple sequence alignments (MSA) of capsid amino acid sequences. Bit-scores and E-values are for the control sequence from each clade used to test the HMMs (**Figure S1**).

LTR Retroelement	MSA (num seqs)	MSA (length)	HMM length	Bit-score	E-value
***Retroviridae* - Class I ERV**	149	294	249	379.7	2.30E-117
***Retroviridae* - Class II ERV**	173	270	213	249.8	1.00E-77
***Retroviridae* - Class III ERV**	17	203	197	306.9	2.50E-95
***Retroviridae* - Spumavirus**	14	175	175	285.4	6.60E-89
***Metaviridae* - Gypsy**	96	220	192	236.2	1.02E-73
***Metaviridae* – ARC**	55	197	149	196.7	1.96E-62
***Pseudoviridae* - Copia**	212	207	174	194.8	3.70E-61
***Belpaoviridae* - BEL**	221	204	180	193.1	1.50E-60
